# In Vivo and In Vitro Antidiabetic and Anti-Inflammatory Properties of Flax (*Linum usitatissimum* L.) Seed Polyphenols

**DOI:** 10.3390/nu13082759

**Published:** 2021-08-11

**Authors:** Hamza Mechchate, Imane Es-safi, Raffaele Conte, Christophe Hano, Amal Amaghnouje, Fatima Zahra Jawhari, Nabil Radouane, Noureddine Bencheikh, Andriy Grafov, Dalila Bousta

**Affiliations:** 1 Laboratory of Biotechnology, Environment, Agri-Food, and Health, Faculty of Sciences Dhar El Mahraz, University Sidi Mohamed Ben Abdellah, P.O. Box 1796, Fez 30000, Morocco; Hamza.mechchate@usmba.ac.ma (H.M.); Amal.amaghnouje@usmba.ac.ma (A.A.); jawhari.fatimazahra@gmail.com (F.Z.J.); Bousta.dalila@usmba.ac.ma (D.B.); 2 Laboratory of Inorganic Chemistry, Department of Chemistry, University of Helsinki, P.O. Box 55, FI-00014 Helsinki, Finland; Andriy.grafov@helsinki.fi; 3 Research Institute on Terrestrial Ecosystems (IRET)—CNR, Via Pietro Castellino 111, 80131 Naples, Italy; Raffaele.conte86@tiscali.it; 4 Laboratoire de Biologie des Ligneux et des Grandes Cultures, INRAE USC1328, Campus Eure et Loir, Orleans University, 45067 Orleans, France; christophe.hano@univ-orleans.fr; 5 Laboratory of Functional Ecology and Environmental Engineering, Sidi Mohamed Ben Abdellah University, P.O. Box 2202, Fez 30000, Morocco; nabil.radouane@usmba.ac.ma; 6 Laboratory of Bioresources, Biotechnology, Ethnopharmacology and Health, Faculty of Sciences, Mohammed First University, Oujda 60000, Morocco; bencheikh_noureddine1718@ump.ac.ma

**Keywords:** flax, flaxseed, polyphenols extraction, diabetes mellitus, antidiabetic, anti-inflammatory, dyslipidemia, carrageenan-induced edema, hemolysis test, LC-MS/MS

## Abstract

Flaxseed is an oilseed (45–50% oil on a dry-weight basis) crop. Its oil has demonstrated multiple health benefits and industrial applications. The goal of this research was to evaluate the antidiabetic and anti-inflammatory potential of the free polyphenol fraction of flax (*Linum usitatissimum* L.) seeds (PLU), based on their use in traditional medicine. Mice with alloxan-induced diabetes were used to study the antidiabetic activity of PLU in vivo, with an oral administration of 25 and 50 mg/kg over 28 days. Measurements of body weight and fasting blood glucose (FBG) were carried out weekly, and biochemical parameters were evaluated. An oral glucose tolerance test was also performed. Inhibitory activities of PLU on α-amylase and α-glucosidase activities were evaluated in vitro. The anti-inflammatory was evaluated in vivo in Wistar rats using the paw edema induction Test by carrageenan, and in vitro using the hemolysis ratio test. PLU administration to diabetic mice during the study period improved their body weight and FBG levels remarkably. In vitro inhibitory activity of digestive enzymes indicated that they may be involved in the proposed mode of action of PLU extract. Qualitative results of PLU revealed the presence of 18 polyphenols. These findings support daily consumption of flaxseed for people with diabetes, and suggest that polyphenols in flaxseed may serve as dietary supplements or novel phytomedicines to treat diabetes and its complications.

## 1. Introduction

Diabetes mellitus represents a global health crisis, with over 693 million cases expected by 2045, according to the International Diabetes Federation (IDF) [1]. Diabetes mellitus is a chronic metabolic disorder affecting the secretion of insulin and its mode of action, resulting in both chronic hyperglycemia and the dysfunctional metabolism of fats, carbohydrates, and protein [2]. Statistics project that one in 11 people will be diabetic, and that one in two adults with diabetes are undiagnosed [3]. This increased prevalence is correlated directly to economic development and urbanization, which have led to drastic changes in lifestyle, characterized by a reduction in physical activity and an increase in obesity [4].

For centuries, herbal medicine has played a significant role in managing diabetes around the world [5,6]. The pharmacological approaches available at present, including oral hypoglycemic agents, have many limitations and side effects, such as hypoglycemia, weight gain, headache, dizziness, and nausea [7], which have led researchers to seek new, safer alternatives [8,9].

*Linum usitatissimum* L. seeds (Linaceae) are commonly called flaxseed or linseed, and is locally called “Zaariat ketan” in Morocco [10]. They are one of the oldest traditional annual cultivated crops in the world [11]. Several studies have indicated that flaxseed is a source of phytochemicals, with potential health benefits [12]. Based on these previous reports, flaxseed oil and fractions have important medicinal activities, including antioxidant, anti-inflammatory, anticancer, antiviral, and bactericidal properties [13]. They can also decrease blood glucose and cholesterol levels [14,15].

The most used part of this seed is its oil, due to its richness in α-linoleic acid (used by the food and cosmetic industries). However, the cold-pressed flaxseed (or linseed) cakes produced as a by-product are also of special interest [16]. The majority of research on the health benefits of the phytochemicals from flax seeds have focused on the fraction from the bound, so-called lignan macromolecule complex [17,18,19,20,21], whereas the unbound polar fraction has received far less attention. In a total valorization strategy based on cracking-type recovery, however, this fraction may be recovered after the extraction of the omega-3-rich oil and before the extraction, combined with hydrolysis, allowing for the recovery of the components from the lignan macromolecule complex, including the well-known lignan secoisolariciresinol diglucoside (SDG). Therefore, the present study aimed to valorize the free polar fraction of the flaxseeds, which also contain polyphenols, by investigating their composition, antidiabetic, antihyperglycemic, and antihyperlipidemic activities in an alloxan-induced diabetic mouse model, and their anti-inflammatory activity in Wistar rats.

## 2. Materials and Methods

### 2.1. Plant Material

*Linum usitatissimum* L. seeds were purchased from an herbalist; the collection location was identified (The latitude: 33.851294 and the longitude: −4.867622). The plant was recognized, validated and authenticated (BPRN57) by a qualified botanist from the LBEAS laboratory at the FSDM, Fez, Morocco.

### 2.2. Animals

Both sexes of Wistar rats (190–230 g) and Swiss albino mice (20–25 g) were provided by the animal center of the department of biology. Animals were housed under a temperature of 23 °C ± 2 °C, a light–dark cycle (12:12), and a humidity of 45–50%. The acclimatization period lasted at least 1 week prior to the beginning of each study. The care and handling of animals followed the international standards of animal use [22]. The procedure was authorized and approved by the institutional animal ethics committee (02/2019/LBEAS).

### 2.3. Preparation of Polyphenolic Extract

The extraction was made following the descriptions found in the following book [23]. *L. usitatissimum* seeds were washed with water, sun dried, then ground into a fine powder. Before beginning the extraction, the powder was defatted (because of the high oil content) as follow: 10 g of the seed powder was washed with hexane (30 mL × 3) (until the organic phase (hexane) is clear). After that, extraction was performed with the use of an ultrasound-assisted extraction apparatus (40 min extraction time and 35 kHz as frequency), 10 g of defatted powder were mixed with 70% methanol. The extract was filtered through Whatman filter paper (No. 5) and concentrated to dryness. The dry extract was mixed with distilled water (DW), and then extracted with 1-butanol (×2) to yield the polyphenolic fraction (PLU). The extract was stored at 4 °C until use.

### 2.4. PLU LC–MS/MS Analysis

The LC-MS/MS analysis was made following a lab developed and optimized method for the sample preparation and the chromatographic analysis.

#### 2.4.1. Samples Preparation

Aliquotes of Samples (80 mg) were treated according to two extraction procedures:

Hydrophilic extraction: The aliquot was treated with 1 mL of ethanol. The eppendorf was vortexed and incubated in a sonicator bath at 45 °C for 60 min.

Lipophilic extraction: The aliquot was treated with 1 mL of acetonitrile plus 500 µL of benzene. The eppendorf was vortexed and incubated in a sonicator bath at 45 °C for 60 min.

#### 2.4.2. Qualitative Analysis

The analysis was conducted using a Shimadzu UHPLC coupled to MS/MS detector (LCMS 8060). Electrospray ionization was used with the MS/MS detector, controlled by a software to allow for the fast switching between low- and high-energy scans (4 V (full scan)—10–60 V (ramping)). The analysis parameters are: 250, 300 and 400 °C for DL, interface and heat block temperature, respectively, 2.9 and 10 L/min for nebulizing and gas flow. The mobile phase consisted of A: acetonitrile and B: water + 0.01% formic acid in a ratio of 5:95, *v*/*v*.

This analysis was performed using a flow injection (no actual chromatographic separation of the compounds), and the detection was made on the basis of the typical fragment of the compound detected by the MS/MS detector. A compound was considered “positive” if its area under the curve was higher in magnitude than that of the blank (see Supplementary Materials File).

### 2.5. Evaluation of Antidiabetic Activity

For a period of four weeks, the anti-diabetic efficacy of PLU was tested in an alloxan-induced diabetic mice model.

#### 2.5.1. Experimental Diabetes Induction

Experimental diabetes was induced using alloxan monohydrate [24]. As the objective was to induce severe diabetes, a dose of 180 mg/kg b.w. of alloxan monohydrate was injected intraperitoneally into animals fasted for 12 h. To prevent hypoglycemic shock, after the induction, 0.2 mL of glucose solution (4 g/L) was administered orally. The first blood glucose measurements were performed after 4 days; animals with BGLs of at least 450 mg/dL were defined as diabetic.

#### 2.5.2. The Fasting Glucose Measurement Experimental Model

Five sets of mice were formed. The first group (A) included normal mice treated with 0.2 mL/day of DW. The latter groups, B, C, D and E, included diabetic mice treated with 0.2 mL/day of DW, 2 mg/kg b.w./day of glibenclamide, 25 mg/kg b.w./day of PLU, and 50 mg/kg b.w./day of PLU, respectively. Treatments were administered daily for 28 days by intragastric gavage. The fasting blood glucose (FBG) levels were first measured 12 h after fasting, then every 7 days following the 28 days of treatment. The glucose oxidase technique was used to measure FBG using a glucometer (Accu-chek, Indianapolis, Indiana) with the blood being collected from the tail vein. The body weight was monitored weekly after the FBG test. At the end of the study period and after anesthesia, the animals were sacrificed via cervical decapitation. To estimate biochemical parameters, blood was collected in a heparinized tube (ASAT, ALAT, urea, creatinine, and plasma lipid profile).

#### 2.5.3. OGTT

Normal mice fasted for 12 h were selected for the OGTT; 1 h prior to the test, all treatments were administered, and, at the T0 of the test, animals were loaded with d-glucose (5 g/kg b.w.). Post drug administration, BGLs were estimated at 30-min intervals from time T0, at T30, T60, and T120 by tail vein puncture using the Accu-chek kit.

#### 2.5.4. In Vitro α-Amylase Inhibitory Activity

This assay was performed following the protocol described in [25]. The substrate solution (0.2 mL) containing starch and the porcine pancreatic amylase solution (0.1 mL with a concentration of 2 units/mL) were added to a PLU solution (0.2 mL) at different concentrations ranging from 1 to 1000 μg/mL.

The reaction took ten minutes to finish at 37 °C, before being stopped by adding 0.5 mL of 50% acetic acid to each tube. The tubes were centrifuged for 5 min at 4 °C at 3000× *rpm* (Hermle LaborTechnik, Wehingen, Germany) GmbH-Z 300 Universal Centrifuge), and the optic density of the supernatant was measured using a spectro-photometer at 595 nm. Acarbose (α-amylase inhibitor) was utilized as a positive control in this test. For each concentration, the experiment was performed three times. The inhibitory activity of the enzyme was determined using the formula:

Activity inhibition = [(A − B)/A] × 100

where A is the control absorbance and B is the sample absorbance. After determining the α-amylase inhibitory activity of each concentration, the IC_50_ values (the concentration required to inhibit 50% of α-amylase) were determined for acarbose and PLU.

#### 2.5.5. In Vitro α-Glucosidase Inhibitory Activity

This assay was performed following the protocol described in [25]. A total of 50 μL of PLU, at different concentrations ranging from 1 to 1000, was prepared and incubated with 10 μL of α-glucosidase (maltase at a concentration of 1 unit/mL) and 125 μL of 0.1 M phosphate buffer for 20 min at 37 °C. 20 μL the substrate (pNPG) at 1 M was prepared and added to the preparation and the whole was incubated for 30 min. A total of 50 μL of Na_2_CO_3_ (0.1 N) was added to stop the reaction. The optical density measurements were carried out at 405 nm. Acarbose was used as the positive control and the experiment was replicated three times for each concentration.

The inhibitory activity of the enzyme was determined using the formula:

Activity inhibition = [(A − B)/A] × 100(1)

where A is the control’s absorbance and B is the sample’s absorbance. After evaluating the inhibitory activity of each concentration against α-glucosidase, the IC_50_ values (concentration necessary to inhibit 50% of α-glucosidase) for acarbose and PLU were calculated.

### 2.6. Anti-Inflammatory Activity Evaluation

#### The Carrageenan-Induced Paw Edema Test

Following the method in [26], the acute anti-inflammatory activity of the PLU extract against induced edema manifested after the 100 μL subplantar injection of carrageenan (1%) into the right hind paws of each rat. Rats were divided into five groups, each with five rats. Group A consisted of rats without induced edema, treated with 0.2 mL of DW. Groups B, C, D, and E consisted of rats with induced edema treated with 0.2 mL of DW, diclofenac (15 mg/kg b.w.), PLU (25 mg/kg b.w.), and PLU (50 mg/kg b.w.), respectively. After 12 h of food deprivation, all groups received treatment 1 h prior to the carrageenan injection. The thickness of the paw was measured before carrageenan injection (T0), and 1, 2, 3, 4, and 6 h after injection. The increase in the thickness and the difference of the thickness were also measured. The following formula was used to calculate the anti-inflammatory activity based on the percentage of inhibition of edema development:

Inhibition (%) = 100 × [1 − (W − X)/(Y − Z)]

W: Paw volume after the carrageenan injection (treated rats);
X: Paw volume before the injection (treated rats);
Y: Paw volume after the carrageenan injection (control rats);
Z: Paw volume before the injection (control rats).

### 2.7. In Vitro Hemolysis Ratio Test

For hemolysis examinations, fresh rat blood was collected from the tail vein. Anticoagulants were applied to fresh blood samples and combined with sterilized physiological saline at a 4:5 ratio. For the negative and positive controls, sterilized physiological saline and DW were used, respectively. Extract solution (0.2 mL) was incubated for 1 h at 37 °C. Later, each tube was centrifuged, and the absorbance of the supernatant was measured at 545 nm. The ratio of hemolysis was calculated as follows:

H (%) = ({D_S_ − D_N_}/{D_pc_ − D_N_}) × 100%

where H is the hemolysis ratio, and D_S_, D_N_, and D_pc_ are the absorbance of samples, negative controls, and positive controls, respectively.

### 2.8. Statistical Analyses

Statistical analyses were performed using the Graph Pad Prism version 8.0. Data were expressed as the means of five replicates ± standard error of the mean. The results were analyzed using analysis of variance and Dunnett’s comparison tests. The results were considered statistically significant at *p* < 0.05.

## 3. Results and Discussion

### 3.1. Qualitative Analysis of Polyphenol Fraction of Linum usitatissimum L. Seeds (PLU)

Liquid chromatography with tandem mass spectrometry (LC–MS/MS) results are detailed in the Supplementary Materials File. Due to the nature of the flow injection analysis, identification was made based on the molecular weight of typical molecule fragments.

Eighteen molecules classified as belonging to polyphenols were identified in the free (i.e., unbound) flaxseed polar fraction (oleocanthal, oleuropein, hesperetin, ursolic acid, amentoflavone, quercetin-3-*O*-glucoside, quercetin-3-*O*-glucuronic acid, kaempferol-3-*O*-glucose, quercetin-3-*O*-hexose-deoxyhexoside, isorhamnetin-3-*O*-rutinoside, isorhamnetin-7-*O*-pentoside, luteolin-7-*O*-glucoside, kaempferol-3-*O*-glucuronic acid, kaempferol-3-*O*-pentoside, kaempferol-3-*O*-hexose-*O*-deohyhexoside, *trans*-cinnamic acid, procyanidin, naringin) (Table 1). Some of these molecules have an approved antidiabetic [27,28,29,30] and anti-inflammatory activities [31,32,33].

### 3.2. Antidiabetic Activity

#### 3.2.1. Effect of PLU Extract on Diabetic Mice’s Glycemia

**Figure 1:**
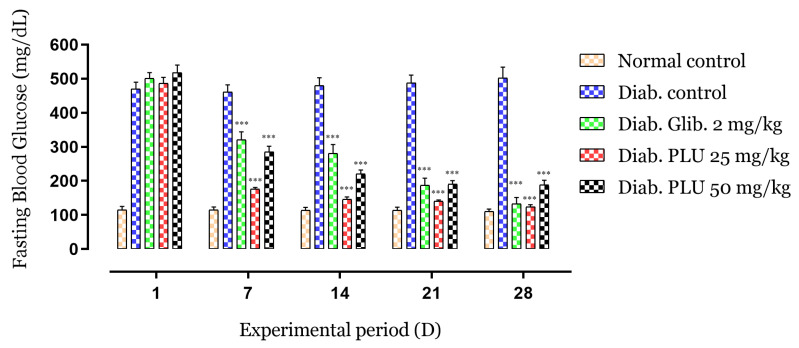
Effects of PLU on the FBG. Values are represented as means ± SD (*n* = 5 mice). *** *p* < 0.001 compared to diabetic controls.

The effects of the repeated oral administration of treatments of alloxan-induced diabetic mice on the blood glucose level (BGL) are shown in Figure 1**.** After 1 week, BGLs were reduced by 64% following treatment with PLU at a dose of 25 mg/kg body weight (b.w.) and by 45% with a dose of 50 mg/kg b.w., or by 36% with glibenclamide, in comparison with the control group (*p* < 0.001). The suppression of hyperglycemia continued significantly (*p* < 0.001) in the second (70%, 58%, and 44% reduction, respectively), third (71%, 63%, and 63% reduction, respectively), and last weeks of treatment (75%, 64%, 73% reduction, respectively) compared to the negative control. These results show a remarkable decrease in and suppression of hyperglycemia in the treated groups with respect to the untreated diabetic mice.

The weekly body weight changes are summarized in Table 2. The untreated diabetic group was marked by a consecutive decrease in body weight during the 4-week period compared to the normal mice. However, as compared to the control group, the diabetic groups treated with PLU for four weeks showed substantial improvements (*p* < 0.001). In a diabetic state, the decline in body weight is highly concomitant with hyperglycemia; the presence of glucose in the blood due to the lack of insulin causes the kidneys to work to eliminate sugar through the urine, which causes weight loss due to dehydration and loss of calories from the lack of sugar-derived energy [34]. Weight loss may also be explained by the excessive catabolism of structural proteins and fat, which are used as a source of energy due to the lack of carbohydrates [35]. Insulin deficiency and resistance may be responsible for the observed catabolism, owing to its significant function as a regulator of protein synthesis and lysis in the skeletal muscle [36]. The oral administration of PLU and glibenclamide to diabetic mice for 4 weeks resulted in an improvement in body weight, which indicates better control of the hyperglycemic state in these mice.

The variations in liver biomarkers ASAT and ALAT are described in Table 3. Significant elevations in the activities of these enzymes were noted in the diabetic control mice compared with the normal control mice (*p* < 0.001); however, the groups treated with PLU (both doses) displayed significantly reduced activities of these enzymes compared to the diabetic control mice (*p* < 0.001). Increased serum levels of these liver biomarkers indicate cellular leakage or hepatocellular membrane damage [37]. One of the proposed causes may be the injection of alloxan [38]. Our results show that PLU exhibits a potential hepatoprotective activity.

Table 3 also describes the variations in kidney biomarkers following the 4 weeks of treatment. As noted, diabetic mice exhibited high levels of urea and creatinine compared to normal control mice (*p* < 0.001). However, diabetic mice treated with PLU displayed significantly decreased creatinine and urea levels in comparison to the diabetic control group (*p* < 0.001). High levels of these kidney biomarkers indicate renal damage [39]. The administration of PLU to the diabetic mice decreased urea and creatinine levels, suggesting a potent preventive action against kidney damage in diabetic conditions.

**Figure 2:**
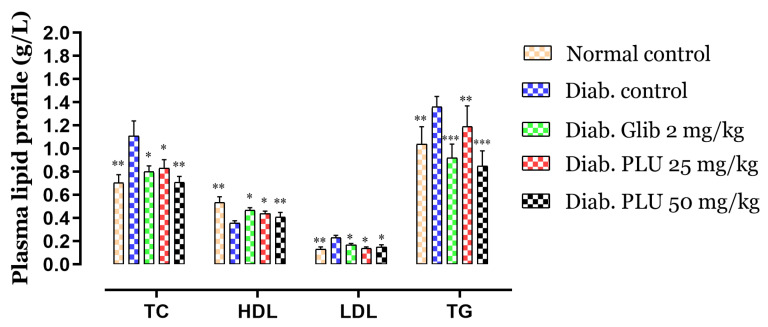
Effects of PLU on plasma lipid profiles. Values are represented as means ± SD (*n* = 5 mice). *** *p* < 0.001, ** *p* < 0.01, and * *p* < 0.05 compared to diabetic controls.

Total cholesterol (TC), triglycerides (TGs), low-density lipoprotein (LDL), and high-density lipoprotein (HDL) levels in the experimental group are shown in Figure 2. TC and TGs were increased by 57% and 31%, respectively, in the diabetic control group in comparison to the normal control mice, while diabetic mice treated with glibenclamide and PLU showed a reduction in plasma TGs (*p* < 0.01 and *p* < 0.001) and TC (*p* < 0.01, *p* < 0.05) compared to the diabetic control mice. However, LDL levels were increased significantly, by 77%, in the diabetic group compared to the normal group. On the administration of PLU, a slight increase, of only 7.7%, was observed for the dose of 25 mg/kg b.w., and 15% for the dose of 50 mg/kg b.w., whereas the glibenclamide group displayed an increase of 33% compared to normal controls. The HDL level was also reduced in the diabetic control group by 33% compared to the normal control mice, versus a reduction of 12%, noted for glibenclamide treatment, and by 18% and 23% for PLU treatments at 25 and 50 mg/kg b.w., respectively.

Dyslipidemia is associated closely with hyperglycemia and is one of the leading cardiovascular disease risk factors. A misfunction of insulin can have a direct impact on lipid metabolism, resulting in hypertriglyceridemia and hypercholesterolemia [40], increasing the secretion of LDL and causing a decrease in HDL levels [41]. After 4 weeks of PLU treatment, abnormal blood lipid levels were reduced, showing that the extract may help with lipid metabolism, and hence avoid diabetes complications, including coronary heart disease and atherosclerosis.

#### 3.2.2. Oral Glucose Tolerance Test (OGTT)

**Figure 3:**
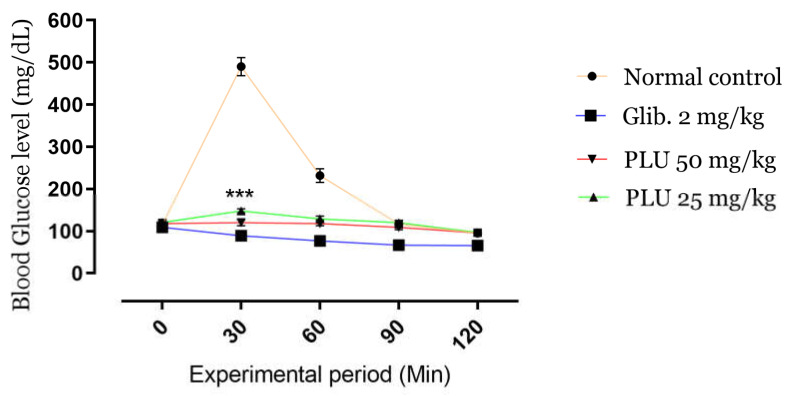
Effects of PLU on BGLs during the OGTT. Values are represented as means ± SD (*n* = 5 mice). *** *p* < 0.001 compared to normal controls.

The kinetics of BGLs during the OGTT are shown in Figure 3. A high-value peak (495 mg/dL) appeared after 30 min of administration of glucose (5 g/kg b.w.) in normal untreated mice; in contrast, the oral administration of PLU, as well as glibenclamide, attenuated BGLs at 30 min significantly (*p* < 0.001), preventing hyperglycemia. BGLs were maintained stably at 60, 90, and 120 min compared to the control groups. The results indicated a remarkable antihyperglycemic activity of the PLU extract at both doses tested.

#### 3.2.3. α-Amylase Inhibitory Effects

Polysaccharide breakdown is facilitated by this enzyme (α-Amylase); it is regarded as one of the most essential enzymes in the digestive process. It is mostly present in pancreatic juice and saliva [8]. Targeting and suppressing this enzyme is one of the potential methods of avoiding increased postprandial blood glucose [42].

**Figure 4:**
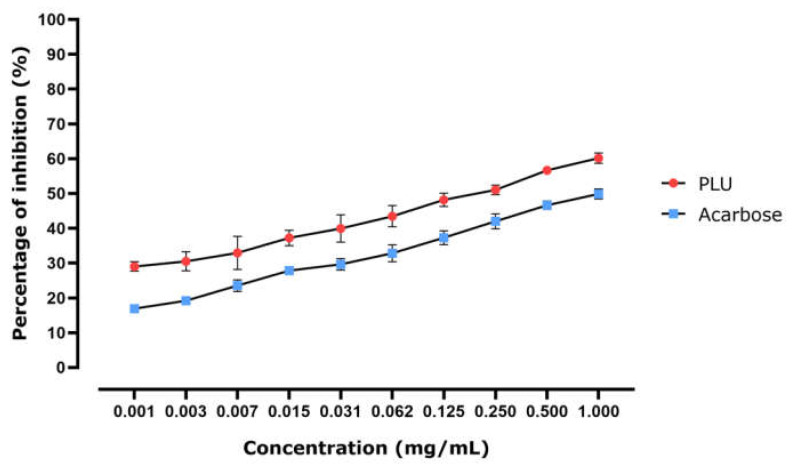
In vitro α-amylase inhibitory activity in response to PLU and acarbose represented as percentage of inhibition of the enzyme activity.

PLU’s ability to inhibit -amylase is illustrated in Figure 4. Enzyme inhibition appears to be dose-dependent, as the PLU dose clearly impacted the quantity of enzyme that was inhibited. The calculated IC_50_ (the concentration required to inhibit 50% of the enzyme) shows that the inhibition potential of acarbose (positive control, 0.717 ± 0.054 mg/mL) was lower than that of PLU, with an IC_50_ of 0.351 ± 0.164 mg/mL. These results indicated that α-amylase inhibitory activity represents one of the modes of action by which PLU lowers the BGL.

#### 3.2.4. α-Glucosidase Inhibitory Effects

The α-glucosidase enzyme, which is located in the small intestine’s mucosal brush border, is similarly crucial for digesting. Its job is to break down and convert complicated carbs into short, easily absorbed sugars. Its blockage is one way to limit glucose absorption and avoid high postprandial blood glucose levels, both of which can slow the progression of diabetes [25].

**Figure 5:**
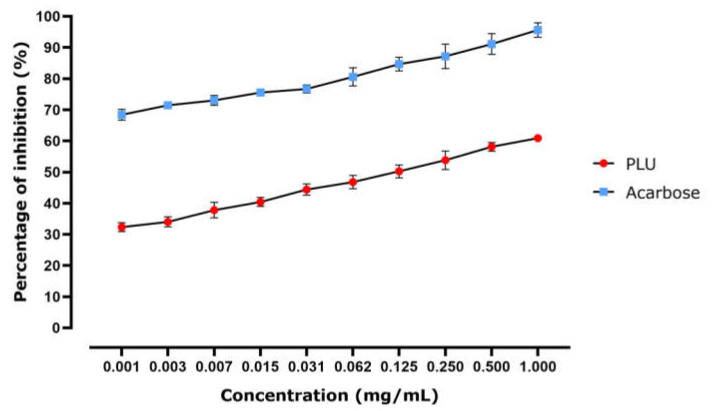
In vitro α-glucosidase inhibitory activity in response to PLU and acarbose represented as percentage of inhibition of the enzyme activity.

Figure 5 demonstrates the α-glucosidase inhibition activity of PLU. The highest dose displayed the highest inhibition efficacy, indicating that the inhibition effect is concentration-dependent. The calculated IC_50_ showed that the inhibition potential of acarbose (IC_50_ = 0.00084 ± 0.0007 mg/mL) was better than that of PLU (IC_50_ = 0.30 ± 0.085 mg/mL), but also suggests that the inhibition of this enzyme may play a role in lowering BGLs.

### 3.3. Anti-Inflammatory Activity

#### 3.3.1. Carrageenan-Induced Paw Edema

**Figure 6:**
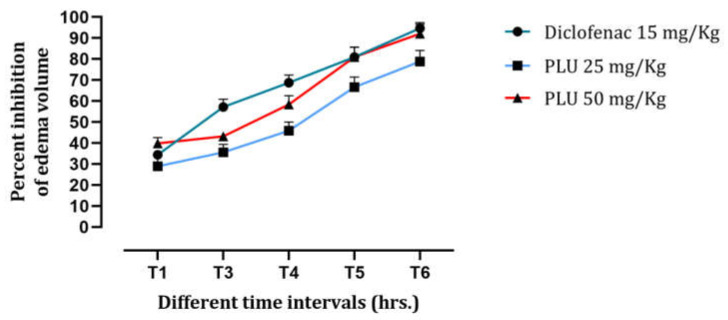
Effects of PLU on the edema development presented as inhibition percentage.

On injecting carrageenan into the hind paw of rats, edema formation was observed, reaching a maximum within 60 min of the injection. Figure 6 shows the inhibition of edema formation in rats following each prescribed treatment. The rats given PLU at a dose of 25 mg/kg b.w. showed an inhibition of 29%, whereas the group treated with 50 mg/kg b.w. showed 40% inhibition, and the group treated with diclofenac showed 34% inhibition. The inhibition continued into the third hour, reaching 36%, 43%, and 57% inhibition, respectively, compared to the untreated controls. The final inhibition, at the conclusion of the experiment, stabilized at 79%, 92%, and 95% inhibition of edema, respectively.

These findings clearly revealed PLU’s anti-inflammatory properties. This standardized model used to study acute inflammation uses carrageenan, a chemical that promotes the release of inflammatory mediators. Carrageenan induces a biphasic response [26,43], in which edema formation and the release of kinins, histamine, and serotonin are observed in the first hour after injection, whereas the second phase is characterized by the release of prostaglandins 2–3 h after the injection. Prostaglandins are defined as the main culprits of acute inflammation [44]. These results demonstrate that PLU has an anti-inflammatory effect in the first and second phases of the carrageenan-induced inflammatory process.

#### 3.3.2. In Vitro Hemolysis Ratio Test

Hemolysis test is performed to evaluate whether red blood cells will remain stable when they come into touch with a foreign substance. According to the national biological safety guidelines, an object is deemed nonhemolytic if the hemolysis rate is lower than 5%. The hemolysis ratio of PLU at various concentrations was tested in vitro by direct contact (Table 4). The results of all samples indicated less than 5% hemolysis, indicating that PLU is nonhemolytic and, therefore, may be safe for use.

## 4. Conclusions

This work attributed, for the first time, the antidiabetic and anti-inflammatory activities of the free (i.e., unbound) polyphenol fraction from *Linum usitatissimum* L. defatted seeds. The biological activities of the flax lignan macromolecule have been extensively investigated; however, here, we show that the far less studied free polar fraction is also of great interest. This work highlighted the remarkable antihyperglycemic and antihyperlipidemic effects of the free polar extract in managing the diabetic status of the experimental animals. The anti-inflammatory activity may also contribute to resolving the complications of diabetes. The extract demonstrated an excellent effect, indicating that the polyphenol fraction of flaxseed represents a potent source of phytochemicals that may be used to manage diabetes and its complications. This study demonstrates that the compounds retained in cold-pressed flaxseed (or linseed) cakes, produced as a by-product following oil extraction, are also of primary interest. The polar free fraction of the flaxseed containing these polyphenols could be further optimized to develop new formulations and phytomedicine.

## Figures and Tables

**Table 1:** Summary of the identified molecules and their abundance estimation in the extract.

Molecule	[M − H]^−^	Relative Abundance in the Extract
Oleocanthal	303.2	+
Oleuropein	539	+++
Hesperetin	301.3	+
Ursolic acid	455	+++
Amentoflavone	537.1	++
Quercetin-3-*O*-glucoside	463.1	+
Quercetin-3-*O*-glucuronic acid	477	+
Kaempferol-3-*O*-glucose	609.1	+
Quercetin-*O*-hexose-*O*-deoxyhexoside	609	+
Isorhamnetin- 3-*O*-rutinoside	623.1	+
Isorhamnetin-7-*O*-pentoside	447.1	+
luteolin 7-*O*-glucoside	447.1	+
Kaempferol-3-*O*-glucuronic acid	461.1	++
Kaempferol-3-*O*-pentose	417.1	+
Kaempferol-3-*O*-hexose-*O*-deohyhexoside	593.1	++
Trans-cinnamic acid	147	+
Procyanidin	577	+
Naringin	579	++

**Table 2:** Body weight changes of normal and diabetic mice during the treatment period.

		Weight	(g)		
	Day 1	Day 7	Day 14	Day 21	Day 28
Norm. control	23.4 ± 1.8	24.7 ± 1.5 *	25.2 ± 1.4 ***	26.9 ± 1.3 ***	27.2 ± 1.5 ***
Diab. control	23.8 ± 1.6	21.7 ± 2.2 ^#^	20.1 ± 2.7 ^###^	19.2 ± 2.5 ^###^	17.7 ± 2.4 ^###^
Diab. Glib 2 mg/kg	24.3 ± 1.6	23.1 ± 1.5 ^ns^	24.2 ± 1.6 **	25.8 ± 1.8 ***	25.9 ± 1.7 ***
Diab. PLU 25 mg/kg	22.8 ± 1.7	23.1 ± 1.6 ^ns^	24.3 ± 1.7 **	25.7 ± 1.8 ***	27.2 ± 1.7 ***
Diab. PLU 50 mg/kg	23.6 ± 2.1	23.5 ± 2.7 ^ns^	24.4 ± 1.9 **	25.4 ± 2.2 ***	26.4 ± 2.4 ***

Values are represented as means ± SD (*n* = 5 mice). ^#^ *p* < 0.05, ^###^ *p* < 0.001 compared to normal controls; * *p* < 0.05, ** *p* < 0.01, and *** *p* < 0.001 compared to diabetic controls; ^ns^ not significant.

**Table 3:** Effects of PLU on urea, creatinine, ASAT, and ALAT levels in alloxan-induced diabetic mice.

	Biomarkers of the Liver		Biomarkers of the Kidney	
Treatment	ASAT (UI/L)	ALAT (UI/L)	Urea (g/L)	Creatinine (mg/L)
Norm. control	45.8 ± 6.1 ***	311 ± 21.2 ***	0.28 ± 0.03 ***	3.4 ± 0.54 ***
Diab. control	134 ± 12.2 ^###^	802 ± 98.3 ^###^	0.63 ± 0.05 ^###^	6.0 ± 0.62 ^###^
Diab. Glib 2 mg/kg	48 ± 4.4 ***	222 ± 15.2 ***	0.28 ± 0.03 ***	4.2 ± 0.35 **
Diab. PLU 25 mg/kg	51 ± 3.1 ***	298 ± 17.7 ***	0.22 ± 0.03 ***	3.8 ± 0.35 ***
Diab. PLU 50 mg/kg	57 ± 4.1 ***	268 ± 18.3 ***	0.30 ± 0.04 ***	4.3 ± 0.32 **

Values are represented as means ± SD (*n* = 5 mice). ^###^ *p* < 0.001 compared to normal controls; ** *p* < 0.01 and *** *p* < 0.001 compared to diabetic controls.

**Table 4:** Hemolysis test results.

Samples	Optical Density (545 nm)	H (%)
Negative control (saline)	0.02246 ± 0.0013	0
Positive control (distilled water)	0.609 ± 0.0120	100
PLU 10 μg/mL	0.042 ± 0.0010	3.40 ± 0.124
PLU 20 μg/mL	0.045 ± 0.0009	3.93 ± 0.098
PLU 50 μg/mL	0.0474 ± 0.00072	4.25 ± 0.309
PLU 100 μg/mL	0.049 ± 0.0009	4.53 ± 0.053
PLU 200 μg/mL	0.0525 ± 0.0062	5.12 ± 0.128

## Data Availability

Data are available upon reasonable request.

## Supplementary Materials

The following are available online at https://www.mdpi.com/article/10.3390/nu13082759/s1, Figure S1: LC/MS-MS data.

## References

[1] Ma Q., Li Y., Li P., Wang M., Wang J., Tang Z., Wang T., Luo L., Wang C., Wang T. (2019). Research progress in the relationship between type 2 diabetes mellitus and intestinal flora. Biomed. Pharmacother..

[2] American Diabetes Association 8 (2019). Obesity Management for the Treatment of Type 2 Diabetes: Standards of Medical Care in Diabetes—2019. Diabetes Care.

[3] Cho N.H., Shaw J.E., Karuranga S., Huang Y., da Rocha Fernandes J.D., Ohlrogge A.W., Malanda B. (2018). IDF Diabetes Atlas: Global estimates of diabetes prevalence for 2017 and projections for 2045. Diabetes Res. Clin. Pract..

[4] Ryan D., Barquera S., Barata Cavalcanti O., Ralston J. (2020). The global pandemic of overweight and obesity: Addressing a twenty-First century multifactorial disease. Handbook of Global Health.

[5] Mechchate H., Ouedrhiri W., Es-safi I., Amaghnouje A., Jawhari F.Z., Bousta D. (2021). Optimization of a New Antihyperglycemic Formulation Using a Mixture of *Linum usitatissimum* L., *Coriandrum sativum* L., and *Olea europaea* var. sylvestris Flavonoids: A Mixture Design Approach. Biologics.

[6] Choudhury H., Pandey M., Hua C.K., Mun C.S., Jing J.K., Kong L., Ern L.Y., Ashraf N.A., Kit S.W., Yee T.S. (2018). An update on natural compounds in the remedy of diabetes mellitus: A systematic review. J. Tradit. Complementary Med..

[7] Chaudhury A., Duvoor C., Reddy Dendi V.S., Kraleti S., Chada A., Ravilla R., Marco A., Shekhawat N.S., Montales M.T., Kuriakose K. (2017). Clinical review of antidiabetic drugs: Implications for type 2 diabetes mellitus management. Front. Endocrinol..

[8] Mechchate H., Es-Safi I., Mohamed Al Kamaly O., Bousta D. (2021). Insight into Gentisic Acid Antidiabetic Potential Using In Vitro and In Silico Approaches. Molecules.

[9] Es-Safi I., Mechchate H., Amaghnouje A., Jawhari F.Z., Bari A., Cerruti P., Avella M., Andriy A., Andriy D. (2020). Medicinal plants used to treat acute digestive system problems in the region of Fez-Meknes in Morocco: An ethnopharmacological survey. Eth. Res. Appl..

[10] Bellakhdar J., Claisse R., Fleurentin J., Younos C. (1991). Repertory of standard herbal drugs in the Moroccan pharmacopoea. J. Ethnopharmacol..

[11] Kiryluk A., Kostecka J. (2020). Pro-environmental and health-promoting grounds for restitution of flax (*Linum usitatissimum* L.) cultivation. J. Ecol. Eng..

[12] Arslanoğlu F., Aytac S. (2020). The important in terms of health of flax (*Linum usitatissimum* L.). Int. J. Life Sci. Biotechnol..

[13] Halligudi N. (2012). Pharmacological properties of flax seeds: A Review. Hygeia JD. Med..

[14] Qureshi J.A., Memon Z., Mirza K.M., Agha S., Saher F., Sunderjee N.F. (2018). Anti hyperglycemic and anti hyperlipidemic activity of linum usitatissimum and glycyrrhiza glabra extracts in streptozotocin-induced diabetic rats. Asian J. Res. Med Pharm. Sci..

[15] Ghule A.E., Jadhav S.S., Bodhankar S.L. (2012). Effect of ethanolic extract of seeds of *Linum usitatissimum* (Linn.) in hyperglycaemia associated ROS production in PBMNCs and pancreatic tissue of alloxan induced diabetic rats. Asian Pac. J. Trop. Dis..

[16] Fliniaux O., Corbin C., Ramsay A., Renouard S., Beejmohun V., Doussot J., Falguières A., Ferroud C., Lamblin F., Lainé E. (2014). Microwave-assisted extraction of herbacetin diglucoside from flax (*Linum usitatissimum* L.) seed cakes and its quantification using an RP-HPLC-UV system. Molecules.

[17] Westcott N.D., Muir A.D. (2003). Flax seed lignan in disease prevention and health promotion. Phytochem. Rev..

[18] Hano C., Renouard S., Molinié R., Corbin C., Barakzoy E., Doussot J., Lamblin F., Lainé E. (2013). Flaxseed (*Linum usitatissimum* L.) extract as well as (+) -secoisolariciresinol diglucoside and its mammalian derivatives are potent inhibitors of α-amylase activity. Bioorganic Med. Chem. Lett..

[19] Ramsay A., Fliniaux O., Quéro A., Molinié R., Demailly H., Hano C., Paetz C., Roscher A., Grand E., Kovensky J. (2017). Kinetics of the incorporation of the main phenolic compounds into the lignan macromolecule during flaxseed development. Food Chem..

[20] Knasmüller S., DeMarini D.M., Johnson I., Gerhäuser C. (2009). Chemoprevention of Cancer and DNA Damage by Dietary Factors.

[21] Hano C., Corbin C., Drouet S., Quéro A., Rombaut N., Savoire R., Molinié R., Thomasset B., Mesnard F., Lainé E. (2017). The lignan (+) -secoisolariciresinol extracted from flax hulls is an effective protectant of linseed oil and its emulsion against oxidative damage. Eur. J. Lipid Sci. Technol..

[22] (2011). National Research Council guide for the care and use of laboratory animals. The National Academies Collection: Reports Funded by National Institutes of Health.

[23] Flavonoids: Chemistry, Biochemistry and Applications. https://www.crcpress.com/Flavonoids-Chemistry-Biochemistry-and-Applications/Andersen-Markham/p/book/9780849320217.

[24] Ighodaro O.M., Adeosun A.M., Akinloye O.A. (2017). Alloxan-induced diabetes, a common model for evaluating the glycemic-control potential of therapeutic compounds and plants extracts in experimental studies. Medicina.

[25] Mechchate H., Es-safi I., Louba A., Alqahtani A.S., Nasr F.A., Noman O.M., Farooq M., Alharbi M.S., Alqahtani A., Bari A. (2021). In vitro alpha-amylase and alpha-glucosidase inhibitory activity and in vivo antidiabetic activity of *Withania frutescens* L. Foliar extract. Molecules.

[26] Mechchate H., Es-Safi I., Amaghnouje A., Boukhira S., Alotaibi A.A., Al-Zharani M., Nasr F.A., Noman O.M., Conte R., Amal E.H.E.Y. (2021). Antioxidant, anti-inflammatory and antidiabetic proprieties of LC-MS/MS identified polyphenols from coriander seeds. Molecules.

[27] Su C., Yang C., Gong M., Ke Y., Yuan P., Wang X., Li M., Zheng X., Feng W. (2019). Antidiabetic activity and potential mechanism of amentoflavone in diabetic mice. Molecules.

[28] Guzmán-Ávila R., Flores-Morales V., Paoli P., Camici G., Ramírez-Espinosa J.J., Cerón-Romero L., Navarrete-Vázquez G., Hidalgo-Figueroa S., Yolanda Rios M., Villalobos-Molina R. (2018). Ursolic Acid Derivatives as Potential Antidiabetic Agents: *In Vitro*, *in Vivo*, and *in Silico* Studies. Drug. Dev. Res..

[29] Haddad P., Eid H. (2017). The antidiabetic potential of quercetin: Underlying mechanisms. Curr. Med. Chem..

[30] Alkhalidy H., Moore W., Wang Y., Luo J., McMillan R., Zhen W., Zhou K., Liu D. (2018). The flavonoid kaempferol ameliorates streptozotocin-induced diabetes by suppressing hepatic glucose production. Molecules.

[31] Muhammad T., Ikram M., Ullah R., Rehman S.U., Kim M.O. (2019). Hesperetin, a citrus flavonoid, attenuates LPS-induced neuroinflammation, apoptosis and memory impairments by modulating TLR4/NF-κB signaling. Nutrients.

[32] Liu C.-M., Ma J.-Q., Xie W.-R., Liu S.-S., Feng Z.-J., Zheng G.-H., Wang A.-M. (2015). Quercetin protects mouse liver against nickel-induced DNA methylation and inflammation associated with the Nrf2/HO-1 and p38/STAT1/NF-κB pathway. Food Chem. Toxicol..

[33] Choi J.S., Islam M.N., Ali M.Y., Kim Y.M., Park H.J., Sohn H.S., Jung H.A. (2014). The effects of C-glycosylation of luteolin on its antioxidant, anti-Alzheimer’s disease, anti-diabetic, and anti-inflammatory activities. Arch. Pharm. Res..

[34] Unwin D.J., Tobin S.D., Murray S.W., Delon C., Brady A.J. (2019). Substantial and sustained improvements in blood pressure, weight and lipid profiles from a carbohydrate restricted diet: An observational study of insulin resistant patients in primary care. Int. J. Environ. Res. Public Health.

[35] Paoli A., Rubini A., Volek J.S., Grimaldi K.A. (2013). Beyond weight loss: A review of the therapeutic uses of very-low-carbohydrate (ketogenic) diets. Eur. J. Clin. Nutr..

[36] Nakashima K., Ishida A., Shimamoto S., Ijiri D., Ohtsuka A. (2019). Insulin stimulation of protein synthesis and mTOR signaling in chick myotube cultures. J. Poult. Sci..

[37] Kumar M., Kaur P., Chandel M., Singh A.P., Jain A., Kaur S. (2017). Antioxidant and hepatoprotective potential of *Lawsonia inermis* L. leaves against 2-acetylaminofluorene induced hepatic damage in male Wistar rats. BMC Complementary Altern. Med..

[38] Ogbeifun H.E., Peters D.E., Monanu M.O. (2020). Ameliorative Effect of Citrullus LANATUS (Water Melon) Seeds on Alloxan Induced Hepato and Nephro Toxicity. Asian J. Adv. Res. Rep..

[39] Luft F.C. (2021). Biomarkers and predicting acute kidney injury. Acta Physiol..

[40] Rao H., Jalali J.A., Johnston T.P., Koulen P. (2021). Emerging Roles of Dyslipidemia and Hyperglycemia in Diabetic Retinopathy: Molecular Mechanisms and Clinical Perspectives. Front. Endocrinol..

[41] Hirano T. (2018). Pathophysiology of diabetic dyslipidemia. J. Atheroscler. Thromb..

[42] Alqahtani A.S., Hidayathulla S., Rehman M.T., ElGamal A.A., Al-Massarani S., Razmovski-Naumovski V., Alqahtani M.S., El Dib R.A., AlAjmi M.F. (2019). Alpha-amylase and alpha-glucosidase enzyme inhibition and antioxidant potential of 3-oxolupenal and katononic acid isolated from Nuxia oppositifolia. Biomolecules.

[43] Es-Safi I., Mechchate H., Amaghnouje A., Calarco A., Boukhira S., Noman O.M., Mothana R.A., Nasr F.A., Bekkari H., Bousta D. (2020). Defatted Hydroethanolic Extract of Ammodaucus leucotrichus Cosson and Durieu Seeds: Antidiabetic and Anti-Inflammatory Activities. Appl. Sci..

[44] Ricciotti E., FitzGerald G.A. (2011). Prostaglandins and inflammation. Arterioscler. Thromb. Vasc. Biol..

