# Supplementary material for: In Vivo and In Vitro Antidiabetic and Anti-Inflammatory Properties of Flax (Linum usitatissimum L.) Seed Polyphenols
Source: Nutrients. 2021 Aug 11;13(8):2759. doi: 10.3390/nu13082759 (PMC8398359; doi:10.3390/nu13082759)
Supplement: Supplementary file 1 [file nutrients-13-02759-s001.zip › nutrients-1295657-supplementary.pdf]

# = Shimadzu LabSolutions Quant. Browser Data Report =

Acquired by	: System Administrator
Data Acquired	: 26/05/2021 11:23:38
Sample Type	: Unknown
Sample Name	: blnk hydrophylic
Sample ID	:
Sample Amount	: 1
Dilution Factor	: 1
Vial#	: 84
Injection Volume	: 0.5 uL
Data Filename	: blnk hydrophylic_007.lcd
Method Filename	: polifenoli screening SIM.lcm
Processed by	: System Administrator
Modified Date	: 26/05/2021 12:36:49

198.9000(+)@1ID#:1 m/z: 198.9000

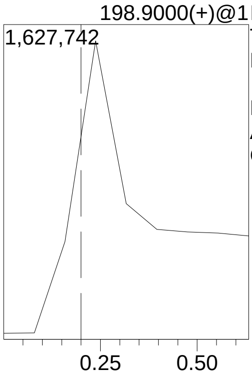

Type:Target
Name:syringic acid SIM

Ret.Time:0.000
Area:0
Conc.:N.D.(Peak)

168.9000(-)@2ID#:2 m/z: 168.9000

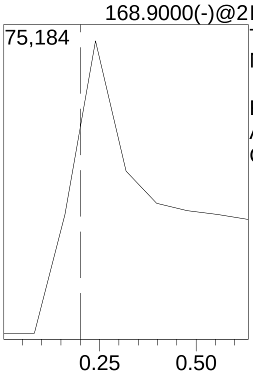

Type:Target
Name:gallic acid SIM

Ret.Time:0.000
Area:0
Conc.:N.D.(Peak)

301.0000(-)@3ID#:3 m/z: 301.0000

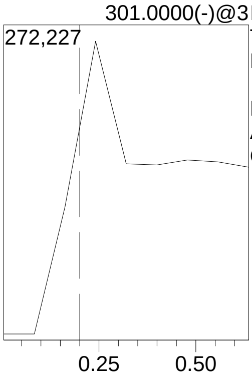

Type:Target
Name:quercetin sim

Ret.Time:0.000
Area:0
Conc.:N.D.(Peak)

162.9000(-)@4ID#:4 m/z: 162.9000

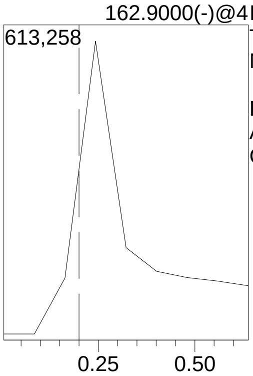

Type:Target
Name:p coumarci acid SIM

Ret.Time:0.000
Area:0
Conc.:N.D.(Peak)

303.2000(-)@5ID#:5 m/z: 303.2000

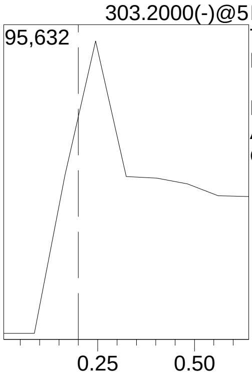

Type:Target
Name:oleochantal SIM

Ret.Time:0.000
Area:0
Conc.:N.D.(Peak)

153.0500(-)@6ID#:6 m/z: 153.0500

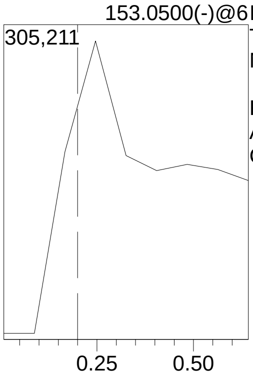

Type:Target
Name:hydroxytyrosol SIM

Ret.Time:0.000
Area:0
Conc.:N.D.(Peak)

193.0000(-)@7ID#:7 m/z: 193.0000

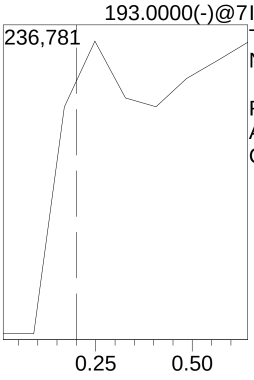

Type:Target
Name:trans ferulic acid SIM

Ret.Time:0.000
Area:0
Conc.:N.D.(Peak)

539.0000(-)@8ID#:8 m/z: 539.0000

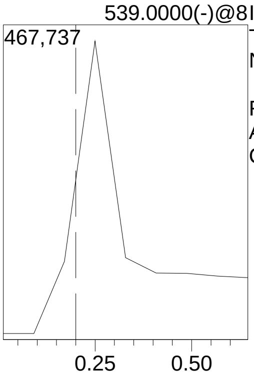

Type:Target
Name:oleuropein SIM

Ret.Time:0.000
Area:0
Conc.:N.D.(Peak)

301.3000(-)@9ID#:9 m/z: 301.3000

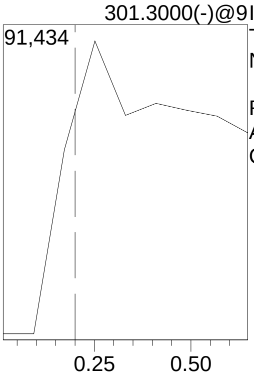

Type:Target
Name:Hesperetin

Ret.Time:0.000
Area:0
Conc.:N.D.(Peak)

312.0000(-)@10ID#:10 m/z: 312.0000

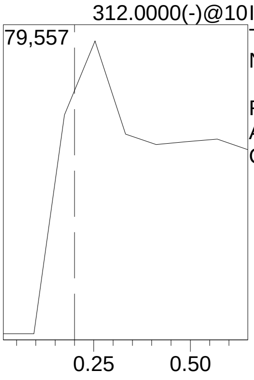

Type:Target
Name:trimethoxyflavone

Ret.Time:0.000
Area:0
Conc.:N.D.(Peak)

271.2000(-)@11ID#:11 m/z: 271.2000

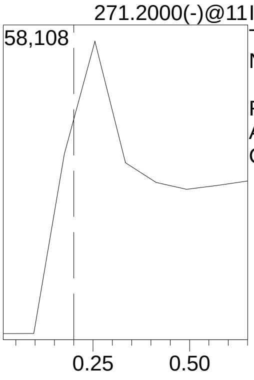

Type:Target
Name:Arbutin

Ret.Time:0.000
Area:0
Conc.:N.D.(Peak)

359.0000(-)@12ID#:12 m/z: 359.0000

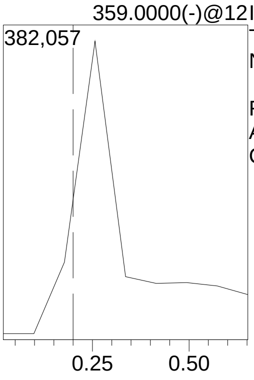

Type:Target
Name:Rosmarinic acid

Ret.Time:0.000
Area:0
Conc.:N.D.(Peak)

455.0000(-)@13ID#:13 m/z: 455.0000

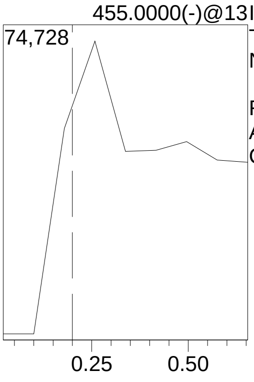

Type:Target
Name:ursolic acid

Ret.Time:0.000
Area:0
Conc.:N.D.(Peak)

269.0000(-)@14ID#:14 m/z: 269.0000

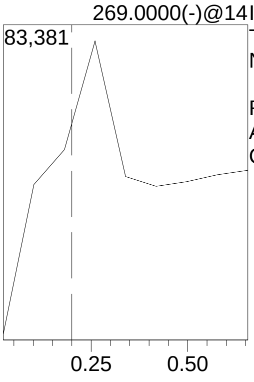

Type:Target
Name:Apigenin

Ret.Time:0.000
Area:0
Conc.:N.D.(Peak)

537.1000(-)@15ID#:15 m/z: 537.1000

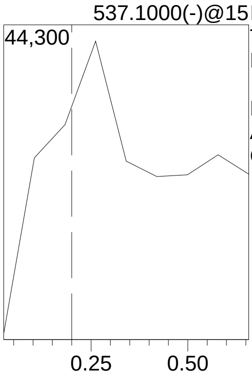

Type:Target
Name:Amentoflavone

Ret.Time:0.000
Area:0
Conc.:N.D.(Peak)

284.9000(-)@16ID#:16 m/z: 284.9000

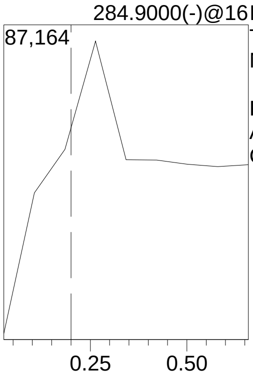

Type:Target
Name:luteoilin

Ret.Time:0.000
Area:0
Conc.:N.D.(Peak)

463.1000(-)@17ID#:17 m/z: 463.1000

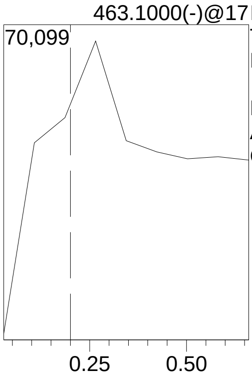

Type:Target
Name:quercetin-3-O-glucoside

Ret.Time:0.000
Area:0
Conc.:N.D.(Peak)

477.0000(-)@18ID#:18 m/z: 477.0000

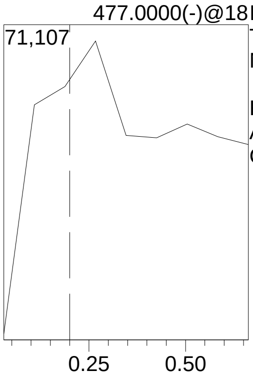

Type:Target
Name:quercetin-3-O- glucuronic acid

Ret.Time:0.000
Area:0
Conc.:N.D.(Peak)

609.1000(-)@19ID#:19 m/z: 609.1000

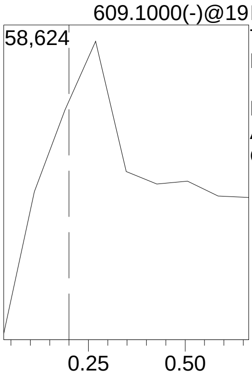

Type:Target
Name:Kaempferol-3-O-glucose

Ret.Time:0.000
Area:0
Conc.:N.D.(Peak)

609.1000(-)@20ID#:20 m/z: 609.1000

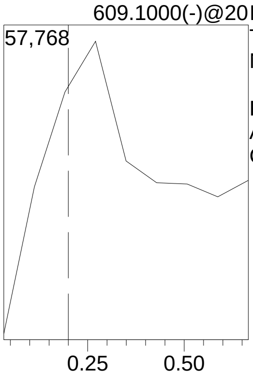

Type:Target
Name:Quercetin-3-O.hexose deoxyhex

Ret.Time:0.000
Area:0
Conc.:N.D.(Peak)

623.1000(-)@21ID#:21 m/z: 623.1000

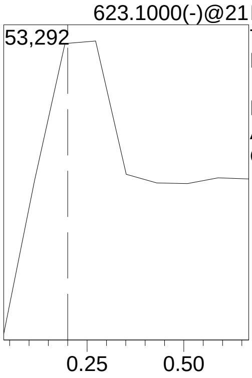

Type:Target
Name:Isorhamnetin- 3-O Rutinoside

Ret.Time:0.000
Area:0
Conc.:N.D.(Peak)

447.1000(-)@22ID#:22 m/z: 447.1000

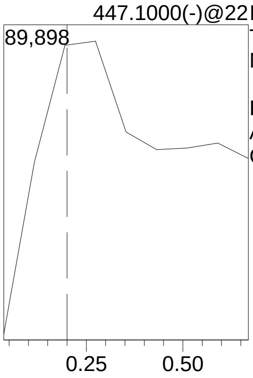

Type:Target
Name:Isorhamnetin-7-O- Pentose

Ret.Time:0.000
Area:0
Conc.:N.D.(Peak)

447.1000(-)@23

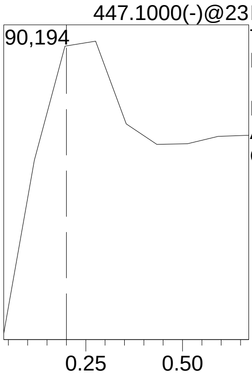

ID#:23 m/z: 447.1000
Type:Target
Name:luteoilin 7-O-glucoside

Ret.Time:0.000
Area:0
Conc.:N.D.(Peak)

461.1000(-)@24

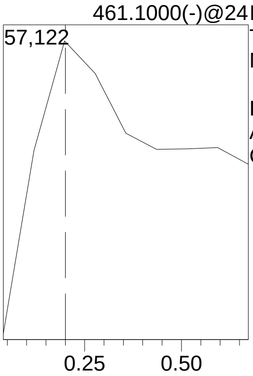

ID#:24 m/z: 461.1000
Type:Target
Name:Kaempferol-3-O-glucuronic acid

Ret.Time:0.000
Area:0
Conc.:N.D.(Peak)

417.1000(-)@25

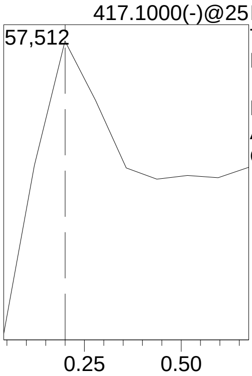

ID#:25 m/z: 417.1000
Type:Target
Name:Kaempferol-3-O-pentose

Ret.Time:0.000
Area:0
Conc.:N.D.(Peak)

593.1000(-)@26

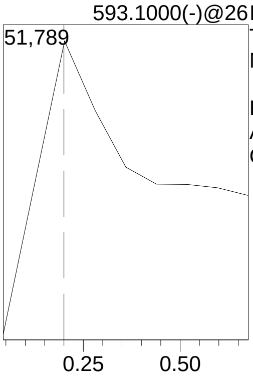

ID#:26 m/z: 593.1000
Type:Target
Name:Kaempferol-3-O-hexose deohyhe

Ret.Time:0.000
Area:0
Conc.:N.D.(Peak)

153.4000(-)@27

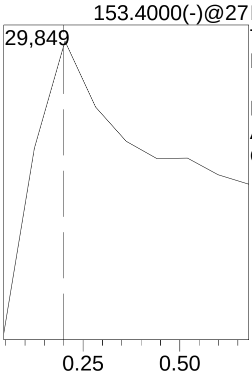

ID#:27 m/z: 153.4000
Type:Target
Name:Tyrosol

Ret.Time:0.000
Area:0
Conc.:N.D.(Peak)

153.0000(-)@28

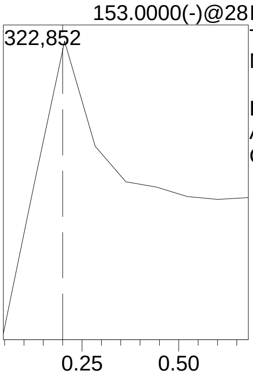

ID#:28 m/z: 153.0000
Type:Target
Name:Protocathecoic acid

Ret.Time:0.000
Area:0
Conc.:N.D.(Peak)

167.0000(-)@29

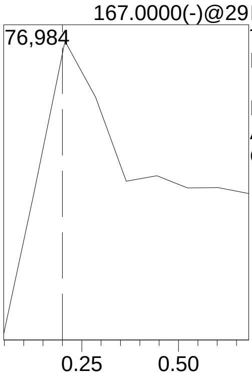

ID#:29 m/z: 167.0000
Type:Target
Name:Vanillic acid

Ret.Time:0.000
Area:0
Conc.:N.D.(Peak)

197.0000(-)@30

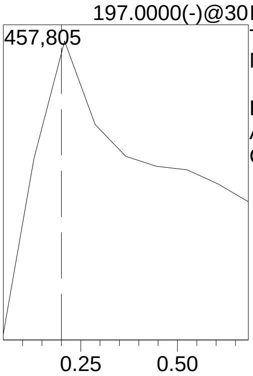

ID#:30 m/z: 197.0000
Type:Target
Name:syringic acid

Ret.Time:0.000
Area:0
Conc.:N.D.(Peak)

137.0000(-)@31ID#:31 m/z: 137.0000

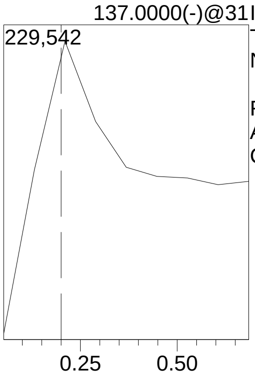

Type:Target
Name:p-hydroxybenzoic\salicilic acid

Ret.Time:0.000
Area:0
Conc.:N.D.(Peak)

153.0000(-)@32ID#:32 m/z: 153.0000

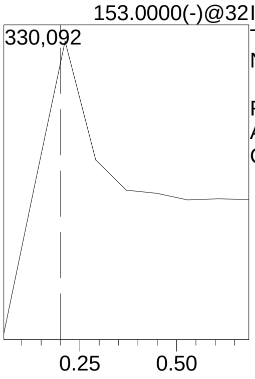

Type:Target
Name:Gentisic acid

Ret.Time:0.000
Area:0
Conc.:N.D.(Peak)

179.0000(-)@33ID#:33 m/z: 179.0000

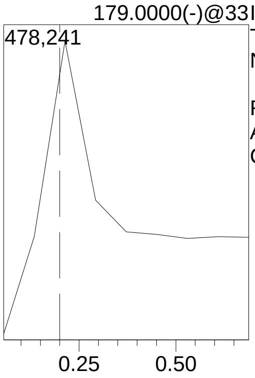

Type:Target
Name:Caffeic acid

Ret.Time:0.000
Area:0
Conc.:N.D.(Peak)

223.0000(-)@34ID#:34 m/z: 223.0000

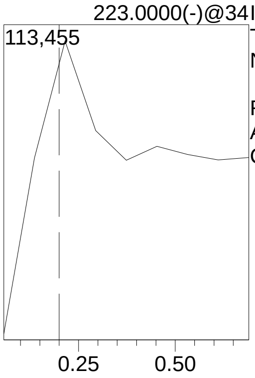

Type:Target
Name:Sinapic acid

Ret.Time:0.000
Area:0
Conc.:N.D.(Peak)

193.0000(-)@35ID#:35 m/z: 193.0000

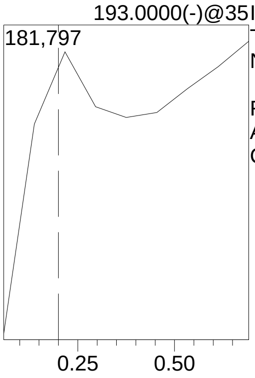

Type:Target
Name:Ferulic acid

Ret.Time:0.000
Area:0
Conc.:N.D.(Peak)

147.0000(-)@36ID#:36 m/z: 147.0000

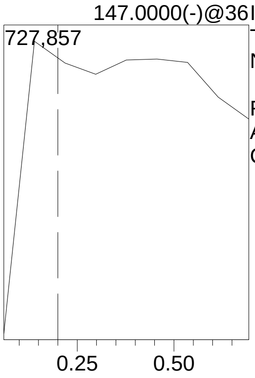

Type:Target
Name:Trans-cinnamic acid

Ret.Time:0.000
Area:0
Conc.:N.D.(Peak)

353.0000(-)@37ID#:37 m/z: 353.0000

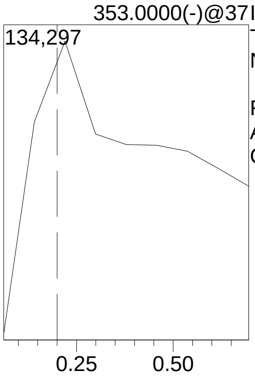

Type:Target
Name:Chlorogenic acid

Ret.Time:0.000
Area:0
Conc.:N.D.(Peak)

289.0000(-)@38ID#:38 m/z: 289.0000

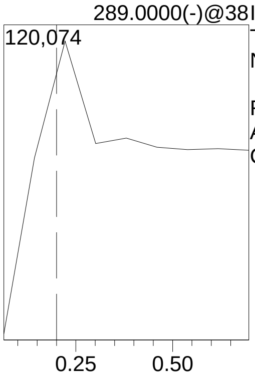

Type:Target
Name:cathechin\epicathechin

Ret.Time:0.000
Area:0
Conc.:N.D.(Peak)

457.0000(-)@39

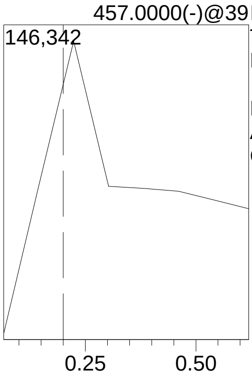

ID#:39 m/z: 457.0000
Type:Target
Name:gallocathechin\epigallocathechin

Ret.Time:0.000
Area:0
Conc.:N.D.(Peak)

305.0000(-)@40

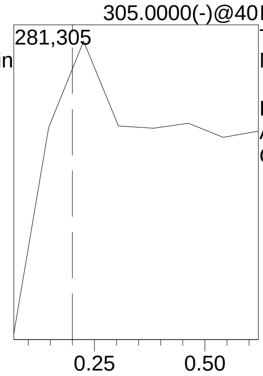

ID#:40 m/z: 305.0000
Type:Target
Name:gallocathechin\epigallocathechin

Ret.Time:0.000
Area:0
Conc.:N.D.(Peak)

441.0000(-)@41

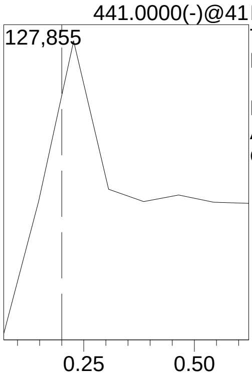

ID#:41 m/z: 441.0000
Type:Target
Name:cathechin gallate

Ret.Time:0.000
Area:0
Conc.:N.D.(Peak)

577.0000(-)@42

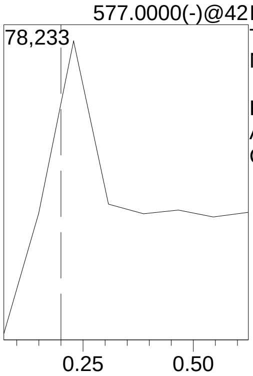

ID#:42 m/z: 577.0000
Type:Target
Name:Procianidin

Ret.Time:0.000
Area:0
Conc.:N.D.(Peak)

317.0000(-)@43

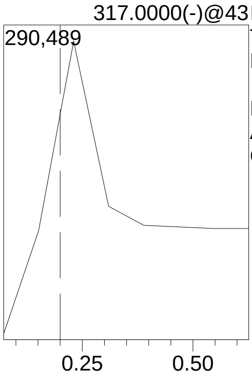

ID#:43 m/z: 317.0000
Type:Target
Name:Myricetin

Ret.Time:0.000
Area:0
Conc.:N.D.(Peak)

285.0000(-)@44

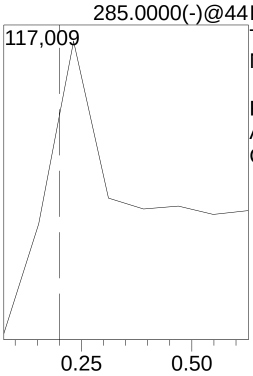

ID#:44 m/z: 285.0000
Type:Target
Name:Kaempeferol

Ret.Time:0.000
Area:0
Conc.:N.D.(Peak)

609.0000(-)@45

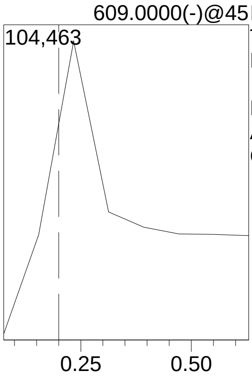

ID#:45 m/z: 609.0000
Type:Target
Name:rutin

Ret.Time:0.000
Area:0
Conc.:N.D.(Peak)

579.0000(-)@46

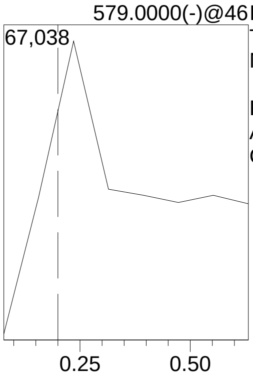

ID#:46 m/z: 579.0000
Type:Target
Name:narigin

Ret.Time:0.000
Area:0
Conc.:N.D.(Peak)

# = Shimadzu LabSolutions Quant. Browser Data Report =

Acquired by	: System Administrator
Data Acquired	: 26/05/2021 11:19:15
Sample Type	: Unknown
Sample Name	: blnk lipophylic
Sample ID	:
Sample Amount	: 1
Dilution Factor	: 1
Vial#	: 81
Injection Volume	: 0.5 uL
Data Filename	: blnk lipophylic_004.lcd
Method Filename	: polifenoli screening SIM.lcm
Processed by	: System Administrator
Modified Date	: 26/05/2021 12:36:49

198.9000(+)@1ID#:1 m/z: 198.9000

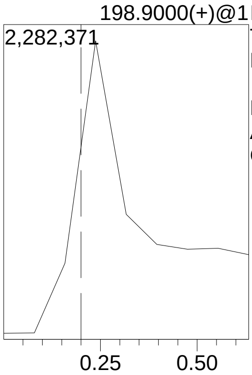

Type:Target
Name:syringic acid SIM

Ret.Time:0.000
Area:0
Conc.:N.D.(Peak)

168.9000(-)@2ID#:2 m/z: 168.9000

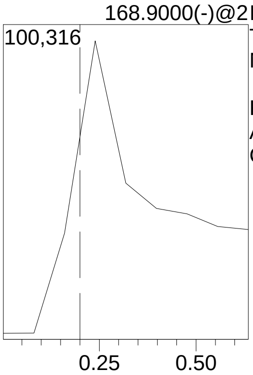

Type:Target
Name:gallic acid SIM

Ret.Time:0.000
Area:0
Conc.:N.D.(Peak)

301.0000(-)@3ID#:3 m/z: 301.0000

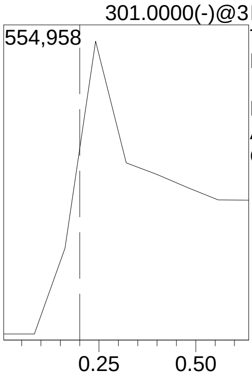

Type:Target
Name:quercetin sim

Ret.Time:0.000
Area:0
Conc.:N.D.(Peak)

162.9000(-)@4ID#:4 m/z: 162.9000

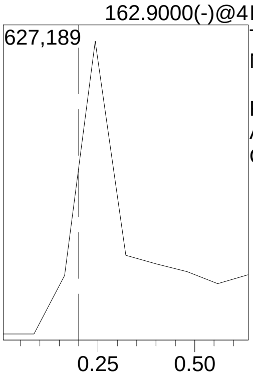

Type:Target
Name:p coumarci acid SIM

Ret.Time:0.000
Area:0
Conc.:N.D.(Peak)

303.2000(-)@5ID#:5 m/z: 303.2000

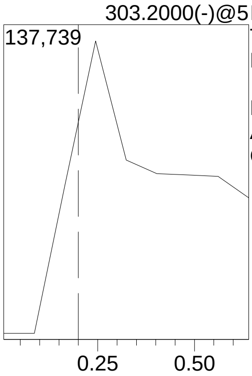

Type:Target
Name:oleochantal SIM

Ret.Time:0.000
Area:0
Conc.:N.D.(Peak)

153.0500(-)@6ID#:6 m/z: 153.0500

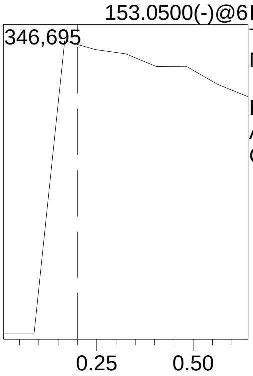

Type:Target
Name:hydroxytyrosol SIM

Ret.Time:0.000
Area:0
Conc.:N.D.(Peak)

193.0000(-)@7ID#:7 m/z: 193.0000

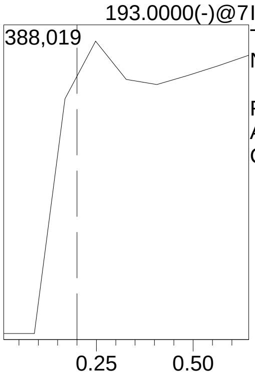

Type:Target
Name:trans ferulic acid SIM

Ret.Time:0.000
Area:0
Conc.:N.D.(Peak)

539.0000(-)@8ID#:8 m/z: 539.0000

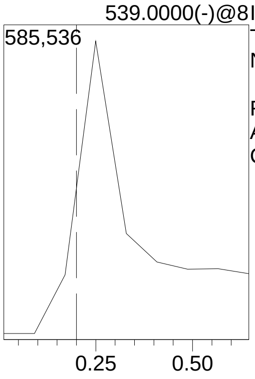

Type:Target
Name:oleuropein SIM

Ret.Time:0.000
Area:0
Conc.:N.D.(Peak)

301.3000(-)@9ID#:9 m/z: 301.3000

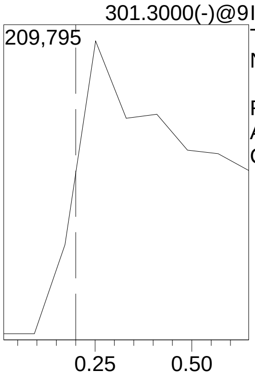

Type:Target
Name:Hesperetin

Ret.Time:0.000
Area:0
Conc.:N.D.(Peak)

312.0000(-)@10ID#:10 m/z: 312.0000

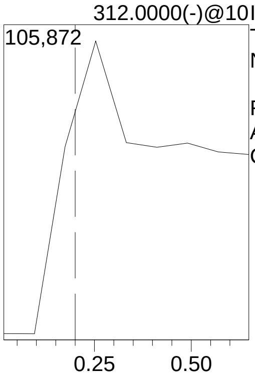

Type:Target
Name:trimethoxyflavone

Ret.Time:0.000
Area:0
Conc.:N.D.(Peak)

271.2000(-)@11ID#:11 m/z: 271.2000

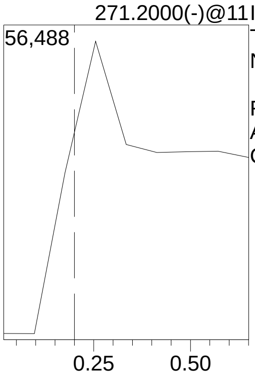

Type:Target
Name:Arbutin

Ret.Time:0.000
Area:0
Conc.:N.D.(Peak)

359.0000(-)@12ID#:12 m/z: 359.0000

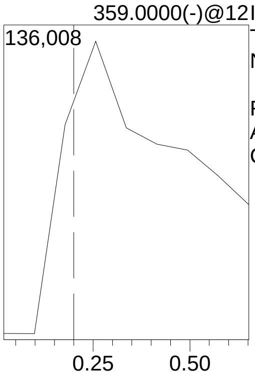

Type:Target
Name:Rosmarinic acid

Ret.Time:0.000
Area:0
Conc.:N.D.(Peak)

455.0000(-)@13ID#:13 m/z: 455.0000

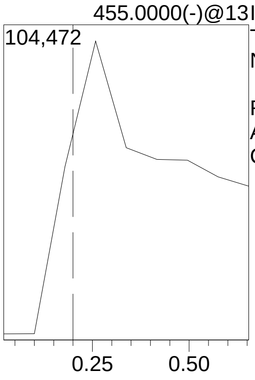

Type:Target
Name:ursolic acid

Ret.Time:0.000
Area:0
Conc.:N.D.(Peak)

269.0000(-)@14ID#:14 m/z: 269.0000

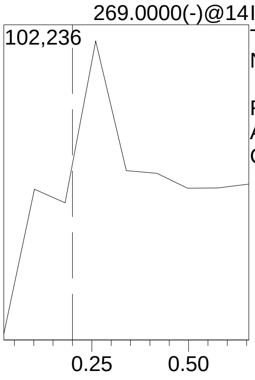

Type:Target
Name:Apigenin

Ret.Time:0.000
Area:0
Conc.:N.D.(Peak)

537.1000(-)@15
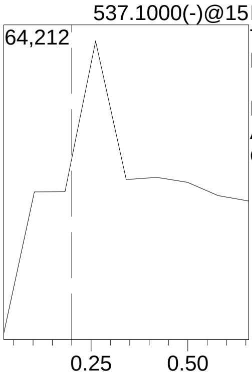

ID#:15 m/z: 537.1000
Type:Target
Name:Amentoflavone

Ret.Time:0.000
Area:0
Conc.:N.D.(Peak)

284.9000(-)@16
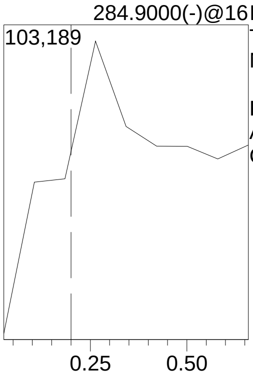

ID#:16 m/z: 284.9000
Type:Target
Name:luteoilin

Ret.Time:0.000
Area:0
Conc.:N.D.(Peak)

463.1000(-)@17
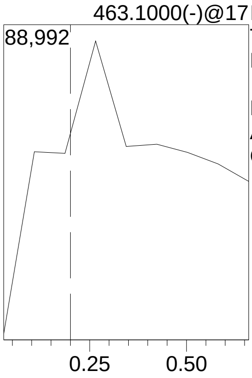

ID#:17 m/z: 463.1000
Type:Target
Name:quercetin-3-O-glucoside

Ret.Time:0.000
Area:0
Conc.:N.D.(Peak)

477.0000(-)@18
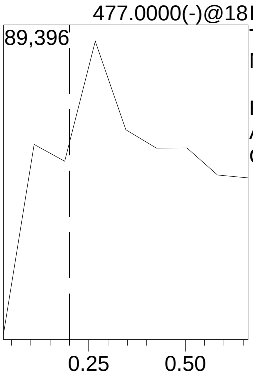

ID#:18 m/z: 477.0000
Type:Target
Name:quercetin-3-O- glucuronic acid

Ret.Time:0.000
Area:0
Conc.:N.D.(Peak)

609.1000(-)@19
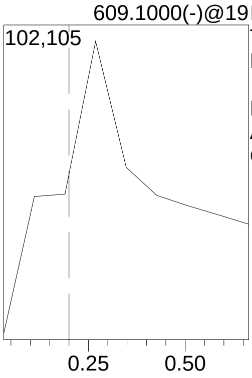

ID#:19 m/z: 609.1000
Type:Target
Name:Kaempferol-3-O-glucose

Ret.Time:0.000
Area:0
Conc.:N.D.(Peak)

609.1000(-)@20
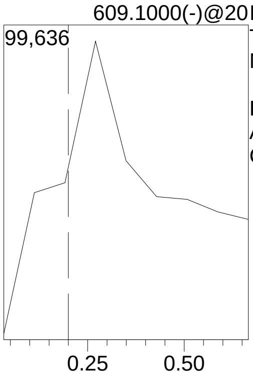

ID#:20 m/z: 609.1000
Type:Target
Name:Quercetin-3-O.hexose deoxyhex

Ret.Time:0.000
Area:0
Conc.:N.D.(Peak)

623.1000(-)@21
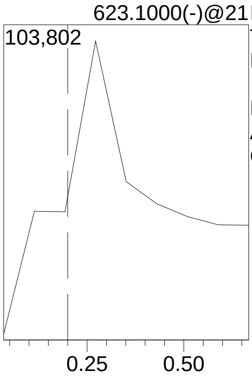

ID#:21 m/z: 623.1000
Type:Target
Name:Isorhamnetin- 3-O Rutinoside

Ret.Time:0.000
Area:0
Conc.:N.D.(Peak)

447.1000(-)@22
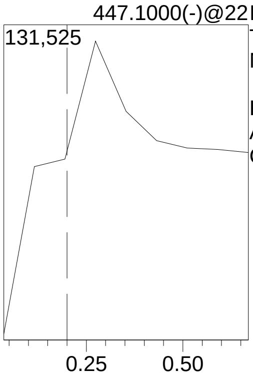

ID#:22 m/z: 447.1000
Type:Target
Name:Isorhamnetin-7-O- Pentose

Ret.Time:0.000
Area:0
Conc.:N.D.(Peak)

447.1000(-)@23 ID#:23 m/z: 447.1000
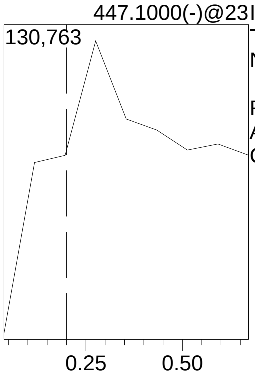

Type:Target
Name:luteoilin 7-O-glucoside

Ret.Time:0.000
Area:0
Conc.:N.D.(Peak)

461.1000(-)@24 ID#:24 m/z: 461.1000
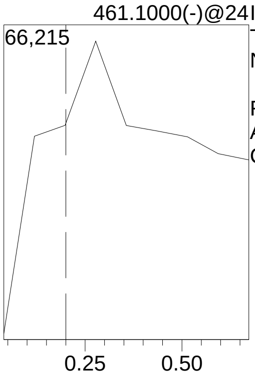

Type:Target
Name:Kaempferol-3-O-glucuronic acid

Ret.Time:0.000
Area:0
Conc.:N.D.(Peak)

417.1000(-)@25 ID#:25 m/z: 417.1000
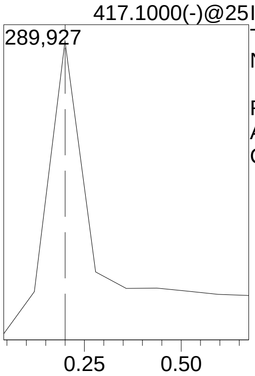

Type:Target
Name:Kaempferol-3-O-pentose

Ret.Time:0.000
Area:0
Conc.:N.D.(Peak)

593.1000(-)@26 ID#:26 m/z: 593.1000
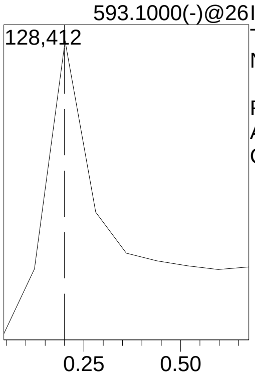

Type:Target
Name:Kaempferol-3-O-hexose deohyhe

Ret.Time:0.000
Area:0
Conc.:N.D.(Peak)

153.4000(-)@27 ID#:27 m/z: 153.4000
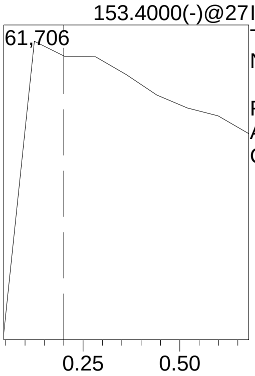

Type:Target
Name:Tyrosol

Ret.Time:0.000
Area:0
Conc.:N.D.(Peak)

153.0000(-)@28 ID#:28 m/z: 153.0000
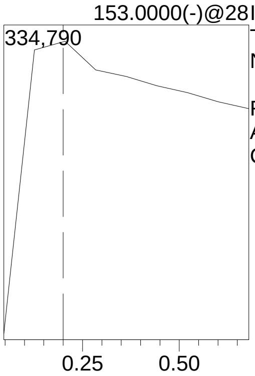

Type:Target
Name:Protocathecoic acid

Ret.Time:0.000
Area:0
Conc.:N.D.(Peak)

167.0000(-)@29 ID#:29 m/z: 167.0000
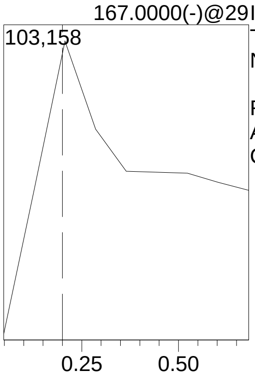

Type:Target
Name:Vanillic acid

Ret.Time:0.000
Area:0
Conc.:N.D.(Peak)

197.0000(-)@30 ID#:30 m/z: 197.0000
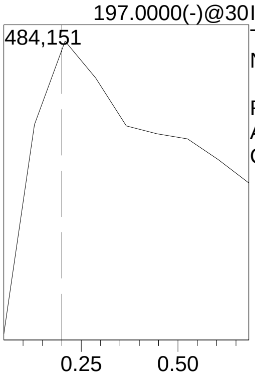

Type:Target
Name:syringic acid

Ret.Time:0.000
Area:0
Conc.:N.D.(Peak)

137.0000(-)@31

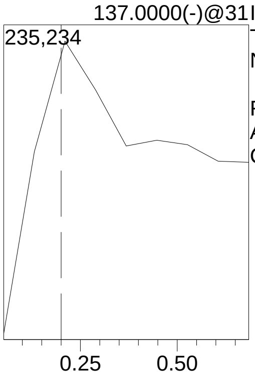

ID#:31 m/z: 137.0000
Type:Target
Name:p-hydroxybenzoic\salicilic acid

Ret.Time:0.000
Area:0
Conc.:N.D.(Peak)

153.0000(-)@32

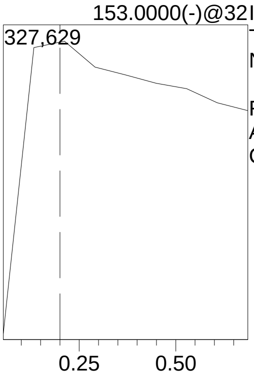

ID#:32 m/z: 153.0000
Type:Target
Name:Gentisic acid

Ret.Time:0.000
Area:0
Conc.:N.D.(Peak)

179.0000(-)@33

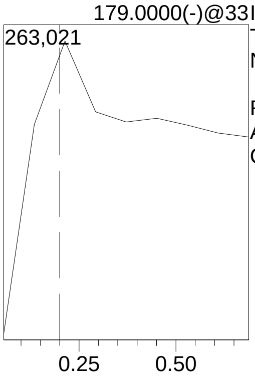

ID#:33 m/z: 179.0000
Type:Target
Name:Caffeic acid

Ret.Time:0.000
Area:0
Conc.:N.D.(Peak)

223.0000(-)@34

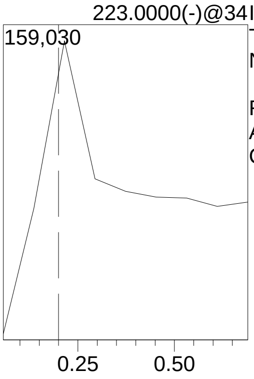

ID#:34 m/z: 223.0000
Type:Target
Name:Sinapic acid

Ret.Time:0.000
Area:0
Conc.:N.D.(Peak)

193.0000(-)@35

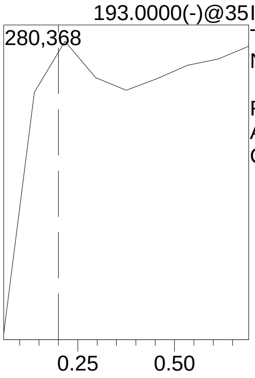

ID#:35 m/z: 193.0000
Type:Target
Name:Ferulic acid

Ret.Time:0.000
Area:0
Conc.:N.D.(Peak)

147.0000(-)@36

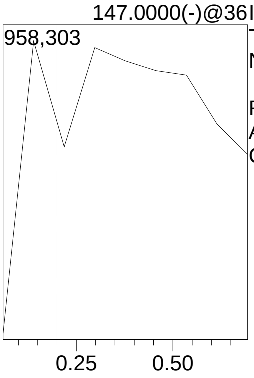

ID#:36 m/z: 147.0000
Type:Target
Name:Trans-cinnamic acid

Ret.Time:0.000
Area:0
Conc.:N.D.(Peak)

353.0000(-)@37

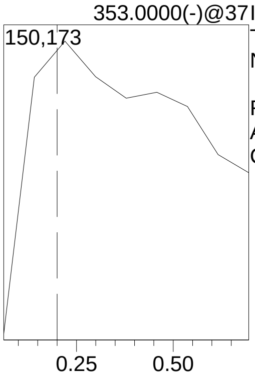

ID#:37 m/z: 353.0000
Type:Target
Name:Chlorogenic acid

Ret.Time:0.000
Area:0
Conc.:N.D.(Peak)

289.0000(-)@38

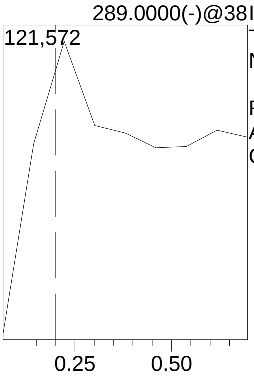

ID#:38 m/z: 289.0000
Type:Target
Name:cathechin\epicathechin

Ret.Time:0.000
Area:0
Conc.:N.D.(Peak)

457.0000(-)@39 ID#:39 m/z: 457.0000
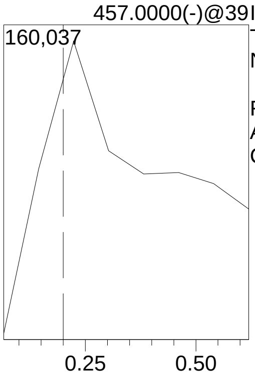

Type:Target
Name:gallocathechin\epigallocathechin

Ret.Time:0.000
Area:0
Conc.:N.D.(Peak)

305.0000(-)@40 ID#:40 m/z: 305.0000
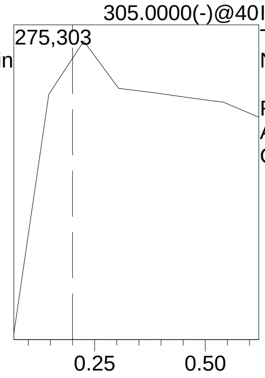

Type:Target
Name:gallocathechin\epigallocathechin

Ret.Time:0.000
Area:0
Conc.:N.D.(Peak)

441.0000(-)@41 ID#:41 m/z: 441.0000
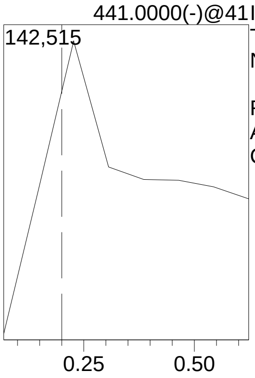

Type:Target
Name:cathechin gallate

Ret.Time:0.000
Area:0
Conc.:N.D.(Peak)

577.0000(-)@42 ID#:42 m/z: 577.0000
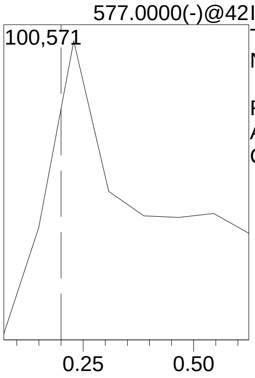

Type:Target
Name:Procianidin

Ret.Time:0.000
Area:0
Conc.:N.D.(Peak)

317.0000(-)@43 ID#:43 m/z: 317.0000
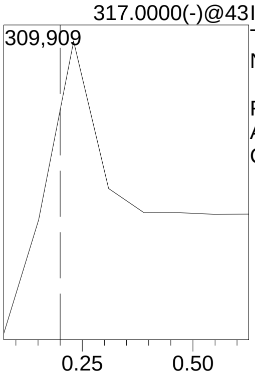

Type:Target
Name:Myricetin

Ret.Time:0.000
Area:0
Conc.:N.D.(Peak)

285.0000(-)@44 ID#:44 m/z: 285.0000
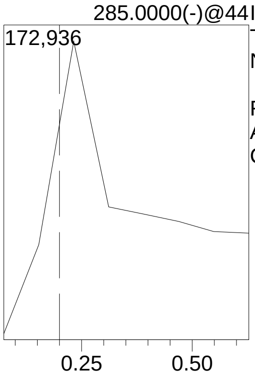

Type:Target
Name:Kaempeferol

Ret.Time:0.000
Area:0
Conc.:N.D.(Peak)

609.0000(-)@45 ID#:45 m/z: 609.0000
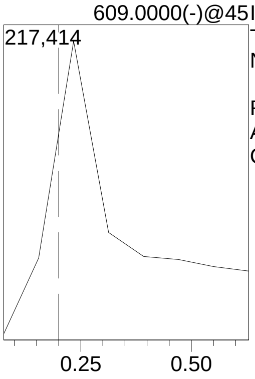

Type:Target
Name:rutin

Ret.Time:0.000
Area:0
Conc.:N.D.(Peak)

579.0000(-)@46 ID#:46 m/z: 579.0000
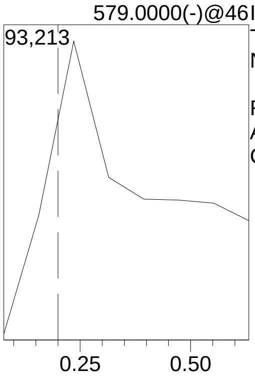

Type:Target
Name:narigin

Ret.Time:0.000
Area:0
Conc.:N.D.(Peak)

# = Shimadzu LabSolutions Quant. Browser Data Report =

Acquired by	: System Administrator
Data Acquired	: 26/05/2021 11:27:57
Sample Type	: Unknown
Sample Name	: hydrophylic extract 13
Sample ID	:
Sample Amount	: 1
Dilution Factor	: 1
Vial#	: 86
Injection Volume	: 0.5 uL
Data Filename	: hydrophylic extract 13_001.lcd
Method Filename	: polifenoli screening SIM.lcm
Processed by	: System Administrator
Modified Date	: 26/05/2021 12:36:50

198.9000(+)@1ID#:1 m/z: 198.9000

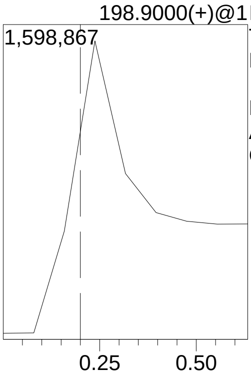

Type:Target
Name:syringic acid SIM

Ret.Time:0.000
Area:0
Conc.:N.D.(Peak)

168.9000(-)@2ID#:2 m/z: 168.9000

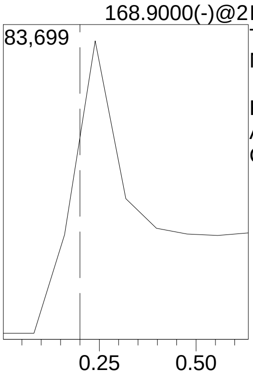

Type:Target
Name:gallic acid SIM

Ret.Time:0.000
Area:0
Conc.:N.D.(Peak)

301.0000(-)@3ID#:3 m/z: 301.0000

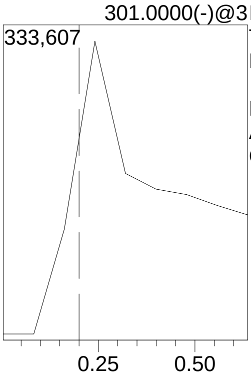

Type:Target
Name:quercetin sim

Ret.Time:0.000
Area:0
Conc.:N.D.(Peak)

162.9000(-)@4ID#:4 m/z: 162.9000

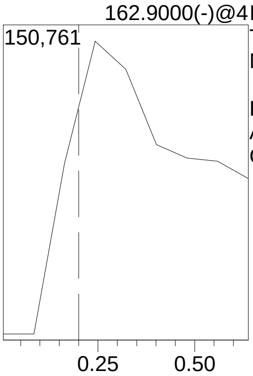

Type:Target
Name:p coumarci acid SIM

Ret.Time:0.000
Area:0
Conc.:N.D.(Peak)

303.2000(-)@5ID#:5 m/z: 303.2000

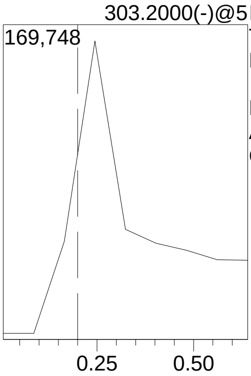

Type:Target
Name:oleochantal SIM

Ret.Time:0.000
Area:0
Conc.:N.D.(Peak)

153.0500(-)@6ID#:6 m/z: 153.0500

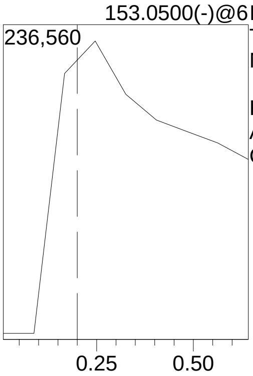

Type:Target
Name:hydroxytyrosol SIM

Ret.Time:0.000
Area:0
Conc.:N.D.(Peak)

193.0000(-)@7ID#:7 m/z: 193.0000

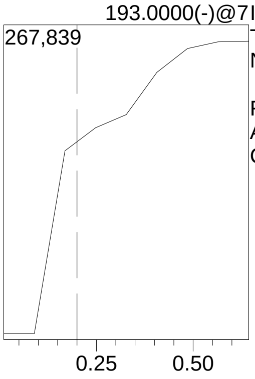

Type:Target
Name:trans ferulic acid SIM

Ret.Time:0.000
Area:0
Conc.:N.D.(Peak)

539.0000(-)@8ID#:8 m/z: 539.0000

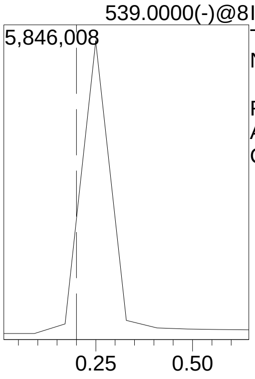

Type:Target
Name:oleuropein SIM

Ret.Time:0.000
Area:0
Conc.:N.D.(Peak)

301.3000(-)@9ID#:9 m/z: 301.3000

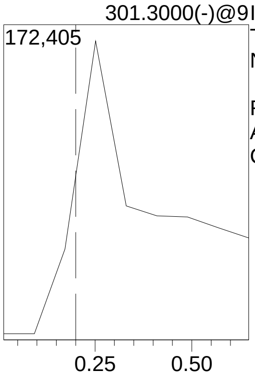

Type:Target
Name:Hesperetin

Ret.Time:0.000
Area:0
Conc.:N.D.(Peak)

312.0000(-)@10ID#:10 m/z: 312.0000

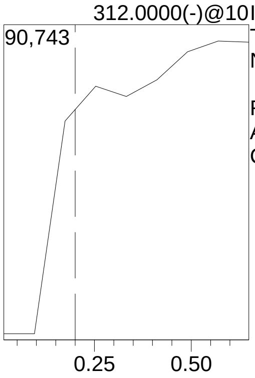

Type:Target
Name:trimethoxyflavone

Ret.Time:0.000
Area:0
Conc.:N.D.(Peak)

271.2000(-)@11ID#:11 m/z: 271.2000

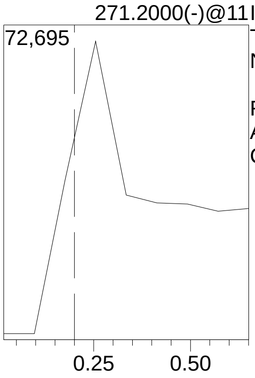

Type:Target
Name:Arbutin

Ret.Time:0.000
Area:0
Conc.:N.D.(Peak)

359.0000(-)@12ID#:12 m/z: 359.0000

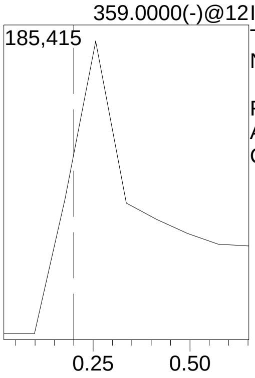

Type:Target
Name:Rosmarinic acid

Ret.Time:0.000
Area:0
Conc.:N.D.(Peak)

455.0000(-)@13ID#:13 m/z: 455.0000

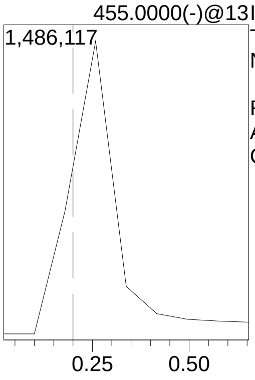

Type:Target
Name:ursolic acid

Ret.Time:0.000
Area:0
Conc.:N.D.(Peak)

269.0000(-)@14ID#:14 m/z: 269.0000

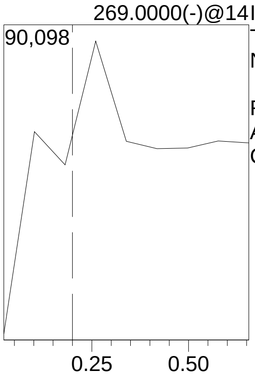

Type:Target
Name:Apigenin

Ret.Time:0.000
Area:0
Conc.:N.D.(Peak)

537.1000(-)@15ID#:15 m/z: 537.1000

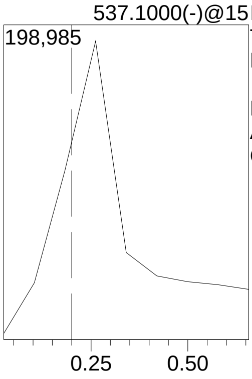

Type:Target
Name:Amentoflavone

Ret.Time:0.000
Area:0
Conc.:N.D.(Peak)

284.9000(-)@16ID#:16 m/z: 284.9000

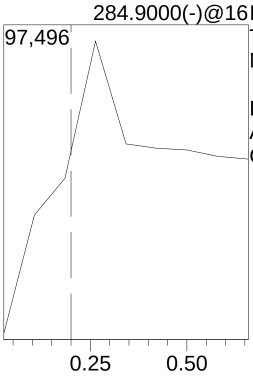

Type:Target
Name:luteoilin

Ret.Time:0.000
Area:0
Conc.:N.D.(Peak)

463.1000(-)@17ID#:17 m/z: 463.1000

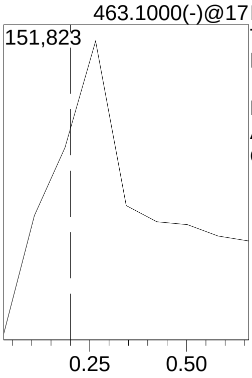

Type:Target
Name:quercetin-3-O-glucoside

Ret.Time:0.000
Area:0
Conc.:N.D.(Peak)

477.0000(-)@18ID#:18 m/z: 477.0000

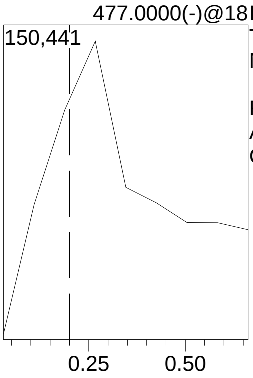

Type:Target
Name:quercetin-3-O- glucuronic acid

Ret.Time:0.000
Area:0
Conc.:N.D.(Peak)

609.1000(-)@19ID#:19 m/z: 609.1000

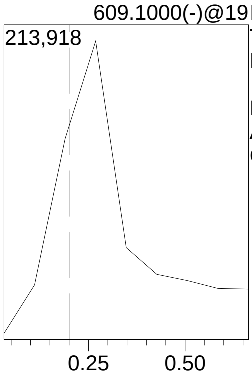

Type:Target
Name:Kaempferol-3-O-glucose

Ret.Time:0.000
Area:0
Conc.:N.D.(Peak)

609.1000(-)@20ID#:20 m/z: 609.1000

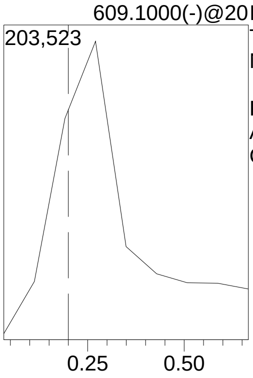

Type:Target
Name:Quercetin-3-O.hexose deoxyhex

Ret.Time:0.000
Area:0
Conc.:N.D.(Peak)

623.1000(-)@21ID#:21 m/z: 623.1000

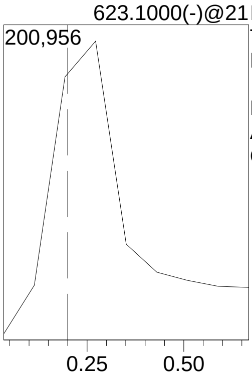

Type:Target
Name:Isorhamnetin- 3-O Rutinoside

Ret.Time:0.000
Area:0
Conc.:N.D.(Peak)

447.1000(-)@22ID#:22 m/z: 447.1000

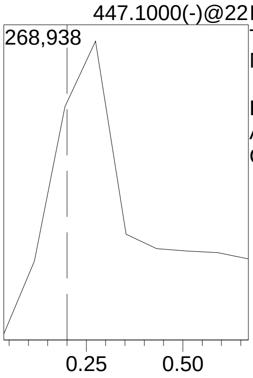

Type:Target
Name:Isorhamnetin-7-O- Pentose

Ret.Time:0.000
Area:0
Conc.:N.D.(Peak)

447.1000(-)@23ID#:23 m/z: 447.1000

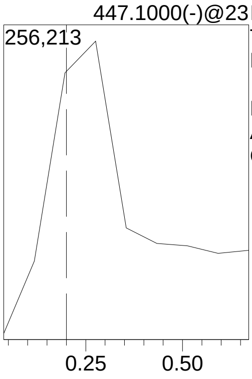

Type:Target
Name:luteoilin 7-O-glucoside

Ret.Time:0.000
Area:0
Conc.:N.D.(Peak)

461.1000(-)@24ID#:24 m/z: 461.1000

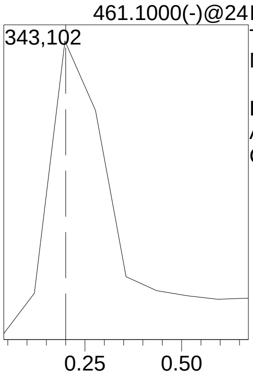

Type:Target
Name:Kaempferol-3-O-glucuronic acid

Ret.Time:0.000
Area:0
Conc.:N.D.(Peak)

417.1000(-)@25ID#:25 m/z: 417.1000

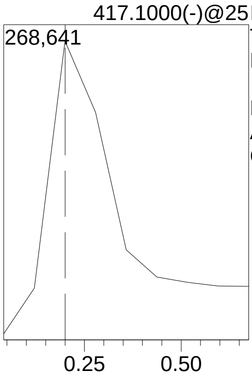

Type:Target
Name:Kaempferol-3-O-pentose

Ret.Time:0.000
Area:0
Conc.:N.D.(Peak)

593.1000(-)@26ID#:26 m/z: 593.1000

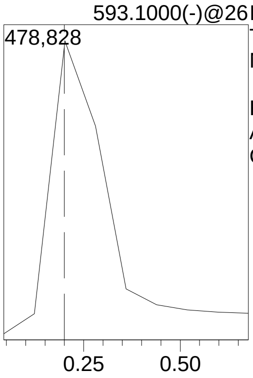

Type:Target
Name:Kaempferol-3-O-hexose deohyhe

Ret.Time:0.000
Area:0
Conc.:N.D.(Peak)

153.4000(-)@27ID#:27 m/z: 153.4000

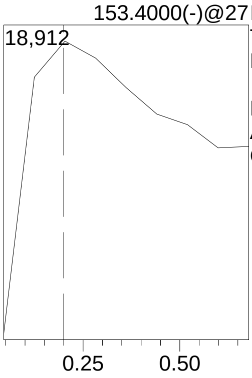

Type:Target
Name:Tyrosol

Ret.Time:0.000
Area:0
Conc.:N.D.(Peak)

153.0000(-)@28ID#:28 m/z: 153.0000

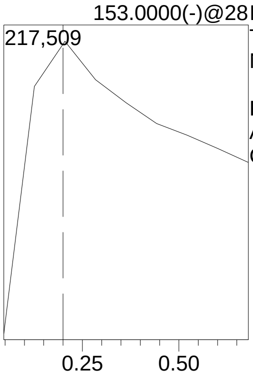

Type:Target
Name:Protocathecoic acid

Ret.Time:0.000
Area:0
Conc.:N.D.(Peak)

167.0000(-)@29ID#:29 m/z: 167.0000

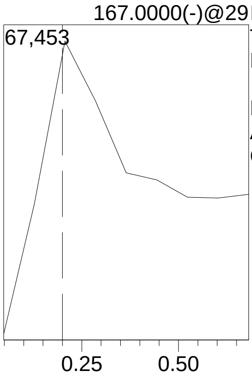

Type:Target
Name:Vanillic acid

Ret.Time:0.000
Area:0
Conc.:N.D.(Peak)

197.0000(-)@30ID#:30 m/z: 197.0000

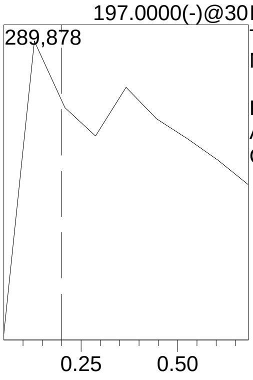

Type:Target
Name:syringic acid

Ret.Time:0.000
Area:0
Conc.:N.D.(Peak)

137.0000(-)@31ID#:31 m/z: 137.0000

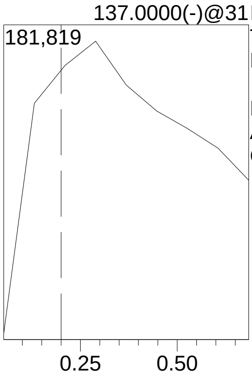

Type:Target
Name:p-hydroxybenzoic\salicilic acid

Ret.Time:0.000
Area:0
Conc.:N.D.(Peak)

153.0000(-)@32ID#:32 m/z: 153.0000

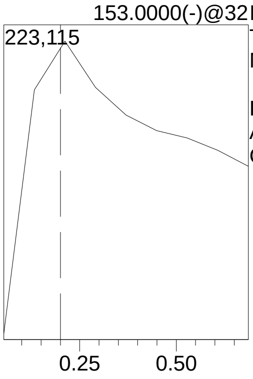

Type:Target
Name:Gentisic acid

Ret.Time:0.000
Area:0
Conc.:N.D.(Peak)

179.0000(-)@33ID#:33 m/z: 179.0000

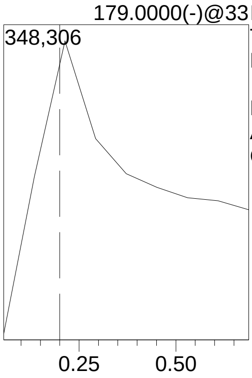

Type:Target
Name:Caffeic acid

Ret.Time:0.000
Area:0
Conc.:N.D.(Peak)

223.0000(-)@34ID#:34 m/z: 223.0000

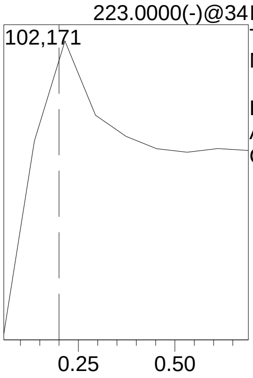

Type:Target
Name:Sinapic acid

Ret.Time:0.000
Area:0
Conc.:N.D.(Peak)

193.0000(-)@35ID#:35 m/z: 193.0000

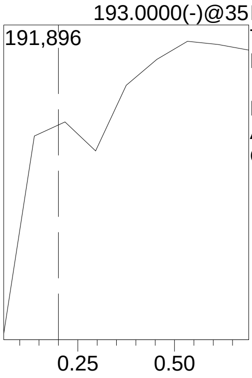

Type:Target
Name:Ferulic acid

Ret.Time:0.000
Area:0
Conc.:N.D.(Peak)

147.0000(-)@36ID#:36 m/z: 147.0000

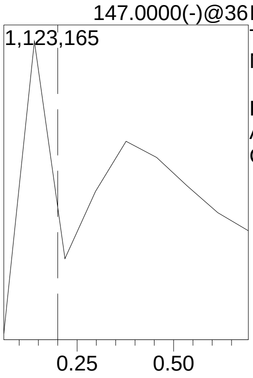

Type:Target
Name:Trans-cinnamic acid

Ret.Time:0.000
Area:0
Conc.:N.D.(Peak)

353.0000(-)@37ID#:37 m/z: 353.0000

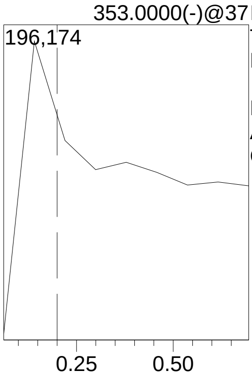

Type:Target
Name:Chlorogenic acid

Ret.Time:0.000
Area:0
Conc.:N.D.(Peak)

289.0000(-)@38ID#:38 m/z: 289.0000

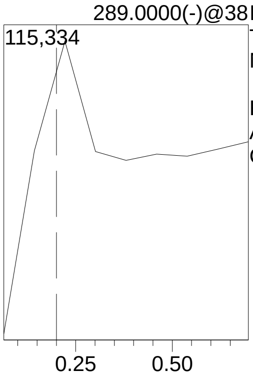

Type:Target
Name:cathechin\epicathechin

Ret.Time:0.000
Area:0
Conc.:N.D.(Peak)

457.0000(-)@39ID#:39 m/z: 457.0000

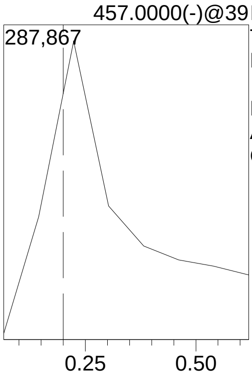

Type:Target
Name:gallocathechin\epigallocathechin

Ret.Time:0.000
Area:0
Conc.:N.D.(Peak)

305.0000(-)@40ID#:40 m/z: 305.0000

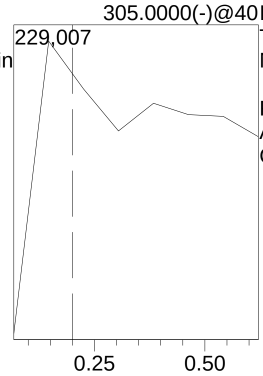

Type:Target
Name:gallocathechin\epigallocathechin

Ret.Time:0.000
Area:0
Conc.:N.D.(Peak)

441.0000(-)@41ID#:41 m/z: 441.0000

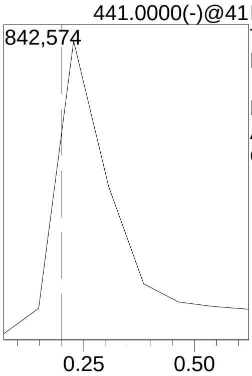

Type:Target
Name:cathechin gallate

Ret.Time:0.000
Area:0
Conc.:N.D.(Peak)

577.0000(-)@42ID#:42 m/z: 577.0000

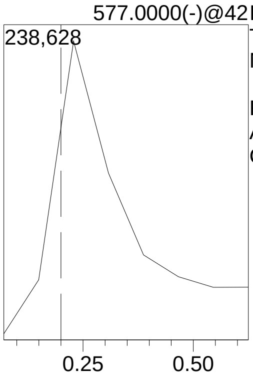

Type:Target
Name:Procianidin

Ret.Time:0.000
Area:0
Conc.:N.D.(Peak)

317.0000(-)@43ID#:43 m/z: 317.0000

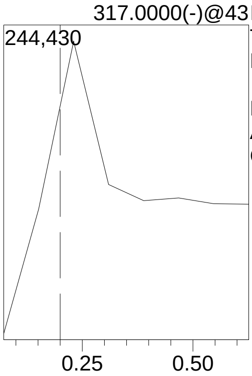

Type:Target
Name:Myricetin

Ret.Time:0.000
Area:0
Conc.:N.D.(Peak)

285.0000(-)@44ID#:44 m/z: 285.0000

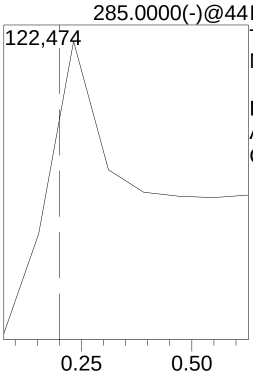

Type:Target
Name:Kaempeferol

Ret.Time:0.000
Area:0
Conc.:N.D.(Peak)

609.0000(-)@45ID#:45 m/z: 609.0000

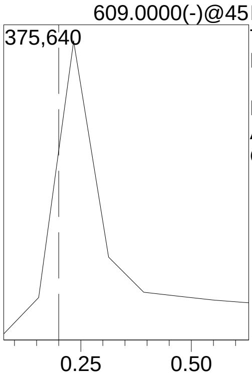

Type:Target
Name:rutin

Ret.Time:0.000
Area:0
Conc.:N.D.(Peak)

579.0000(-)@46ID#:46 m/z: 579.0000

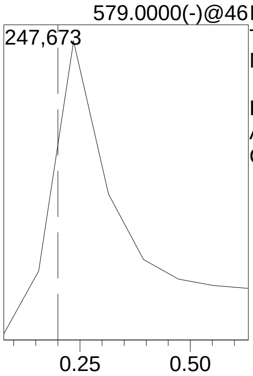

Type:Target
Name:narigin

Ret.Time:0.000
Area:0
Conc.:N.D.(Peak)

# = Shimadzu LabSolutions Quant. Browser Data Report =

Acquired by	: System Administrator
Data Acquired	: 26/05/2021 11:22:10
Sample Type	: Unknown
Sample Name	: lipophylic extract 13
Sample ID	:
Sample Amount	: 1
Dilution Factor	: 1
Vial#	: 83
Injection Volume	: 0.5 uL
Data Filename	: lipophylic extract 13_006.lcd
Method Filename	: polifenoli screening SIM.lcm
Processed by	: System Administrator
Modified Date	: 26/05/2021 12:36:49

198.9000(+)@1ID#:1 m/z: 198.9000

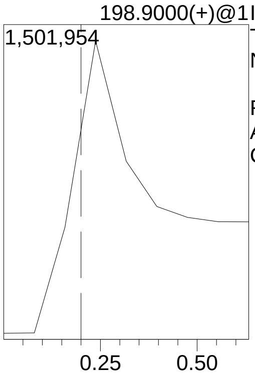

Type:Target
Name:syringic acid SIM

Ret.Time:0.000
Area:0
Conc.:N.D.(Peak)

168.9000(-)@2ID#:2 m/z: 168.9000

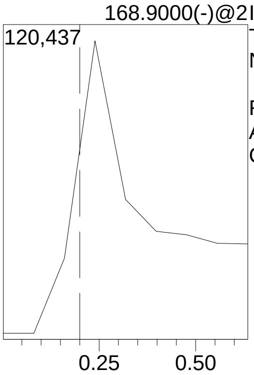

Type:Target
Name:gallic acid SIM

Ret.Time:0.000
Area:0
Conc.:N.D.(Peak)

301.0000(-)@3ID#:3 m/z: 301.0000

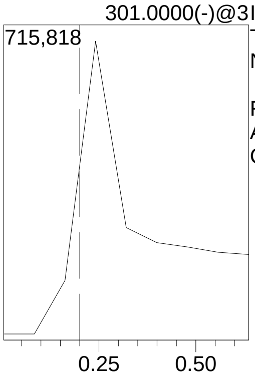

Type:Target
Name:quercetin sim

Ret.Time:0.000
Area:0
Conc.:N.D.(Peak)

162.9000(-)@4ID#:4 m/z: 162.9000

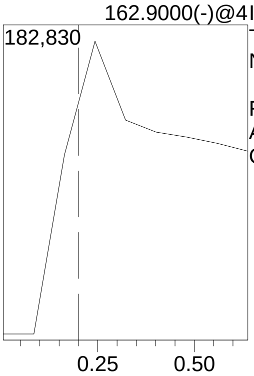

Type:Target
Name:p coumarci acid SIM

Ret.Time:0.000
Area:0
Conc.:N.D.(Peak)

303.2000(-)@5ID#:5 m/z: 303.2000

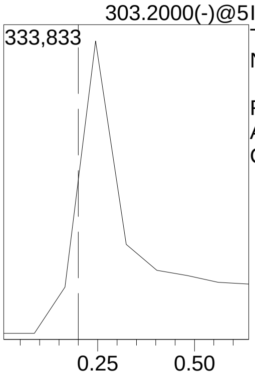

Type:Target
Name:oleochantal SIM

Ret.Time:0.000
Area:0
Conc.:N.D.(Peak)

153.0500(-)@6ID#:6 m/z: 153.0500

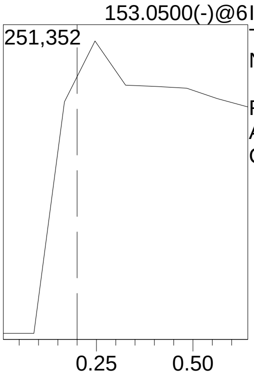

Type:Target
Name:hydroxytyrosol SIM

Ret.Time:0.000
Area:0
Conc.:N.D.(Peak)

193.0000(-)@7ID#:7 m/z: 193.0000

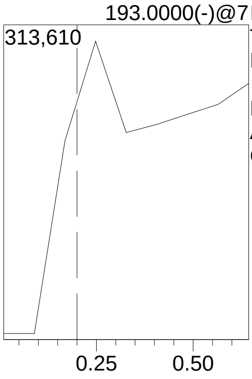

Type:Target
Name:trans ferulic acid SIM

Ret.Time:0.000
Area:0
Conc.:N.D.(Peak)

539.0000(-)@8ID#:8 m/z: 539.0000

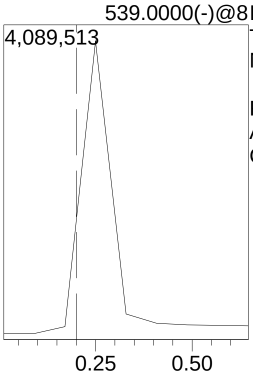

Type:Target
Name:oleuropein SIM

Ret.Time:0.000
Area:0
Conc.:N.D.(Peak)

301.3000(-)@9ID#:9 m/z: 301.3000

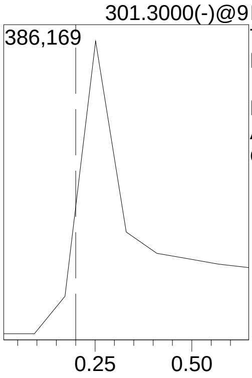

Type:Target
Name:Hesperetin

Ret.Time:0.000
Area:0
Conc.:N.D.(Peak)

312.0000(-)@10ID#:10 m/z: 312.0000

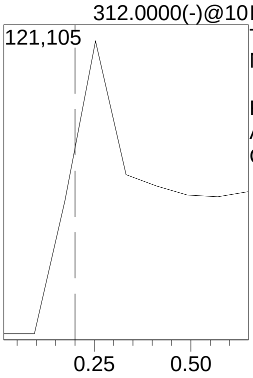

Type:Target
Name:trimethoxyflavone

Ret.Time:0.000
Area:0
Conc.:N.D.(Peak)

271.2000(-)@11ID#:11 m/z: 271.2000

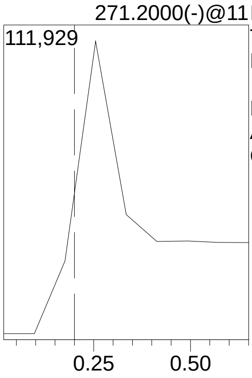

Type:Target
Name:Arbutin

Ret.Time:0.000
Area:0
Conc.:N.D.(Peak)

359.0000(-)@12ID#:12 m/z: 359.0000

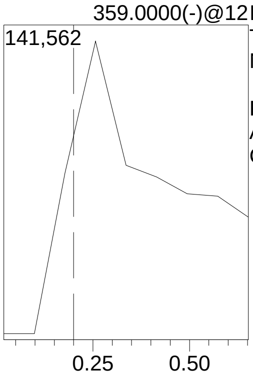

Type:Target
Name:Rosmarinic acid

Ret.Time:0.000
Area:0
Conc.:N.D.(Peak)

455.0000(-)@13ID#:13 m/z: 455.0000

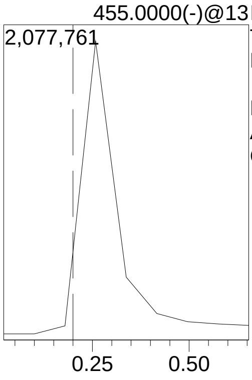

Type:Target
Name:ursolic acid

Ret.Time:0.000
Area:0
Conc.:N.D.(Peak)

269.0000(-)@14ID#:14 m/z: 269.0000

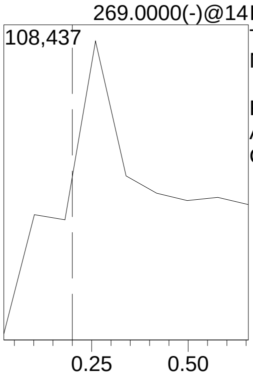

Type:Target
Name:Apigenin

Ret.Time:0.000
Area:0
Conc.:N.D.(Peak)

537.1000(-)@15ID#:15 m/z: 537.1000

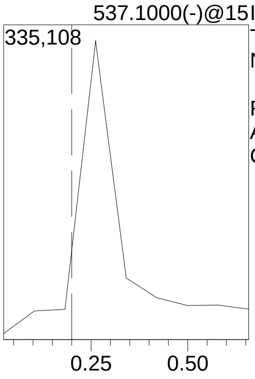

Type:Target
Name:Amentoflavone

Ret.Time:0.000
Area:0
Conc.:N.D.(Peak)

284.9000(-)@16ID#:16 m/z: 284.9000

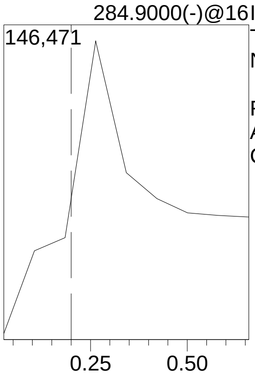

Type:Target
Name:luteoilin

Ret.Time:0.000
Area:0
Conc.:N.D.(Peak)

463.1000(-)@17ID#:17 m/z: 463.1000

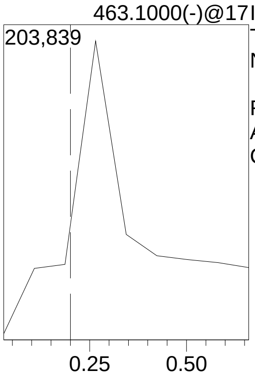

Type:Target
Name:quercetin-3-O-glucoside

Ret.Time:0.000
Area:0
Conc.:N.D.(Peak)

477.0000(-)@18ID#:18 m/z: 477.0000

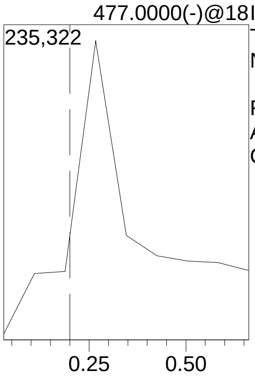

Type:Target
Name:quercetin-3-O- glucuronic acid

Ret.Time:0.000
Area:0
Conc.:N.D.(Peak)

609.1000(-)@19ID#:19 m/z: 609.1000

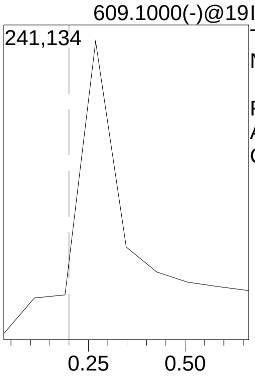

Type:Target
Name:Kaempferol-3-O-glucose

Ret.Time:0.000
Area:0
Conc.:N.D.(Peak)

609.1000(-)@20ID#:20 m/z: 609.1000

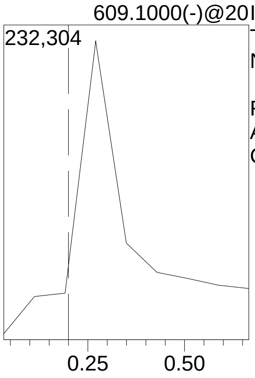

Type:Target
Name:Quercetin-3-O.hexose deoxyhex

Ret.Time:0.000
Area:0
Conc.:N.D.(Peak)

623.1000(-)@21ID#:21 m/z: 623.1000

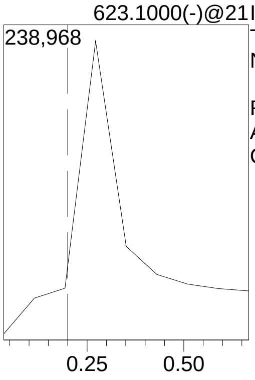

Type:Target
Name:Isorhamnetin- 3-O Rutinoside

Ret.Time:0.000
Area:0
Conc.:N.D.(Peak)

447.1000(-)@22ID#:22 m/z: 447.1000

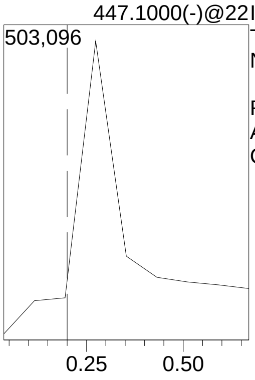

Type:Target
Name:Isorhamnetin-7-O- Pentose

Ret.Time:0.000
Area:0
Conc.:N.D.(Peak)

447.1000(-)@23ID#:23 m/z: 447.1000

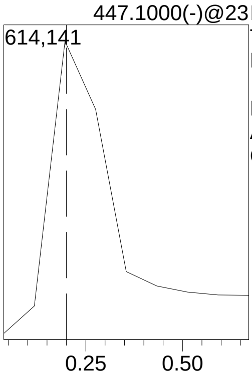

Type:Target
Name:luteoilin 7-O-glucoside

Ret.Time:0.000
Area:0
Conc.:N.D.(Peak)

461.1000(-)@24ID#:24 m/z: 461.1000

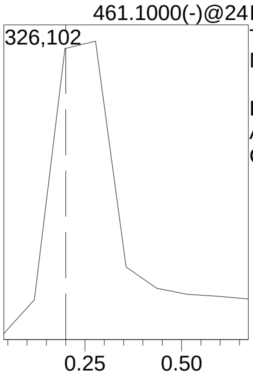

Type:Target
Name:Kaempferol-3-O-glucuronic acid

Ret.Time:0.000
Area:0
Conc.:N.D.(Peak)

417.1000(-)@25ID#:25 m/z: 417.1000

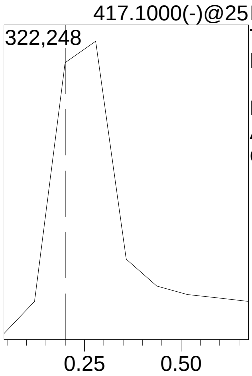

Type:Target
Name:Kaempferol-3-O-pentose

Ret.Time:0.000
Area:0
Conc.:N.D.(Peak)

593.1000(-)@26ID#:26 m/z: 593.1000

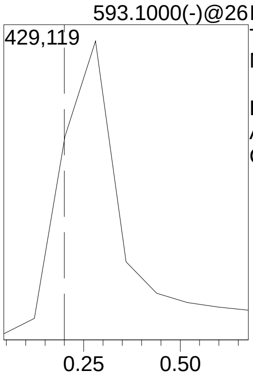

Type:Target
Name:Kaempferol-3-O-hexose deohyh[illegible]

Ret.Time:0.000
Area:0
Conc.:N.D.(Peak)

153.4000(-)@27ID#:27 m/z: 153.4000

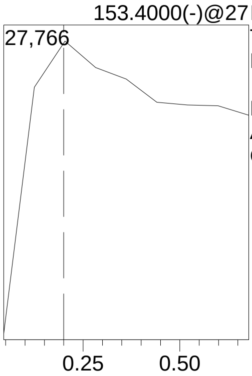

Type:Target
Name:Tyrosol

Ret.Time:0.000
Area:0
Conc.:N.D.(Peak)

153.0000(-)@28ID#:28 m/z: 153.0000

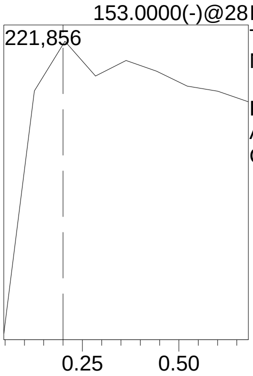

Type:Target
Name:Protocathecoic acid

Ret.Time:0.000
Area:0
Conc.:N.D.(Peak)

167.0000(-)@29ID#:29 m/z: 167.0000

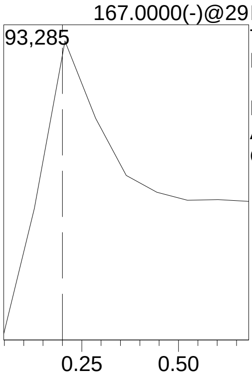

Type:Target
Name:Vanillic acid

Ret.Time:0.000
Area:0
Conc.:N.D.(Peak)

197.0000(-)@30ID#:30 m/z: 197.0000

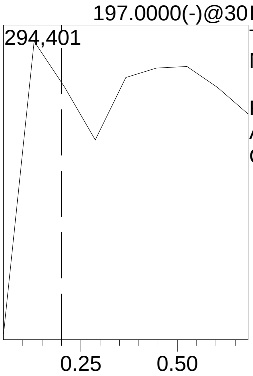

Type:Target
Name:syringic acid

Ret.Time:0.000
Area:0
Conc.:N.D.(Peak)

137.0000(-)@31ID#:31 m/z: 137.0000

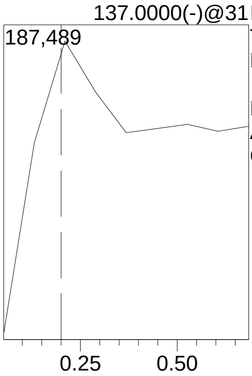

Type:Target
Name:p-hydroxybenzoic\salicilic acid

Ret.Time:0.000
Area:0
Conc.:N.D.(Peak)

153.0000(-)@32ID#:32 m/z: 153.0000

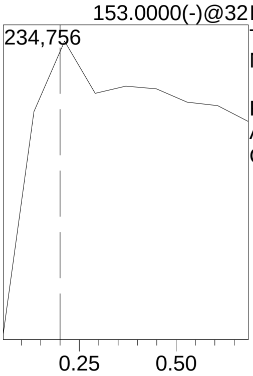

Type:Target
Name:Gentisic acid

Ret.Time:0.000
Area:0
Conc.:N.D.(Peak)

179.0000(-)@33ID#:33 m/z: 179.0000

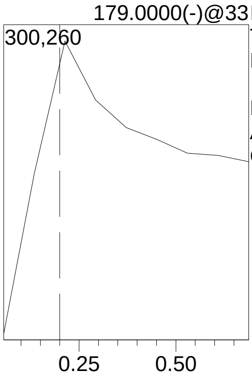

Type:Target
Name:Caffeic acid

Ret.Time:0.000
Area:0
Conc.:N.D.(Peak)

223.0000(-)@34ID#:34 m/z: 223.0000

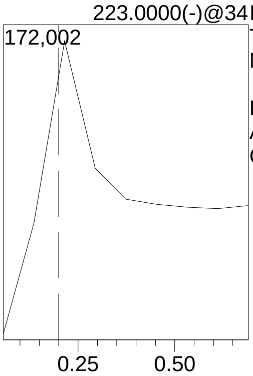

Type:Target
Name:Sinapic acid

Ret.Time:0.000
Area:0
Conc.:N.D.(Peak)

193.0000(-)@35ID#:35 m/z: 193.0000

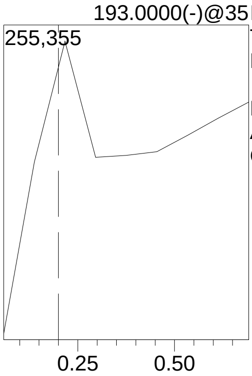

Type:Target
Name:Ferulic acid

Ret.Time:0.000
Area:0
Conc.:N.D.(Peak)

147.0000(-)@36ID#:36 m/z: 147.0000

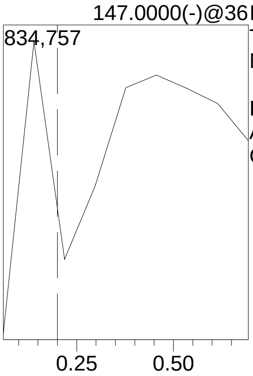

Type:Target
Name:Trans-cinnamic acid

Ret.Time:0.000
Area:0
Conc.:N.D.(Peak)

353.0000(-)@37ID#:37 m/z: 353.0000

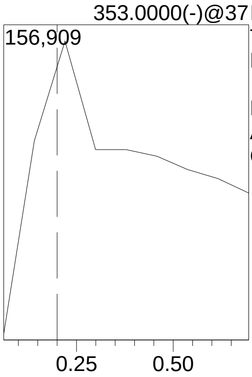

Type:Target
Name:Chlorogenic acid

Ret.Time:0.000
Area:0
Conc.:N.D.(Peak)

289.0000(-)@38ID#:38 m/z: 289.0000

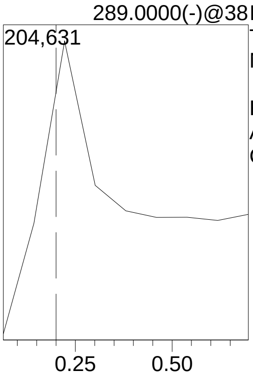

Type:Target
Name:cathechin\epicathechin

Ret.Time:0.000
Area:0
Conc.:N.D.(Peak)

457.0000(-)@39

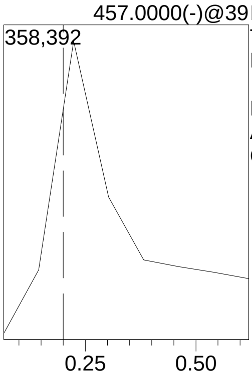

ID#:39 m/z: 457.0000
Type:Target
Name:gallocathechin\epigallocathechin

Ret.Time:0.000
Area:0
Conc.:N.D.(Peak)

305.0000(-)@40

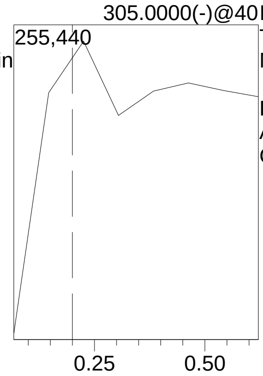

ID#:40 m/z: 305.0000
Type:Target
Name:gallocathechin\epigallocathechin

Ret.Time:0.000
Area:0
Conc.:N.D.(Peak)

441.0000(-)@41

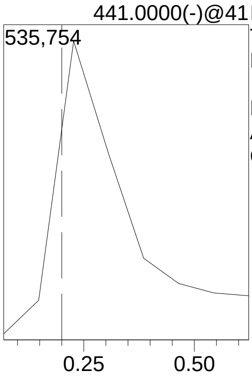

ID#:41 m/z: 441.0000
Type:Target
Name:cathechin gallate

Ret.Time:0.000
Area:0
Conc.:N.D.(Peak)

577.0000(-)@42

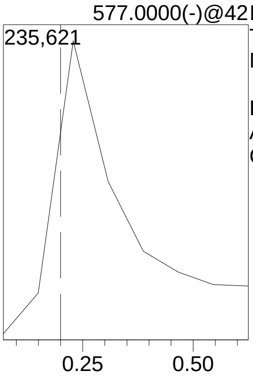

ID#:42 m/z: 577.0000
Type:Target
Name:Procianidin

Ret.Time:0.000
Area:0
Conc.:N.D.(Peak)

317.0000(-)@43

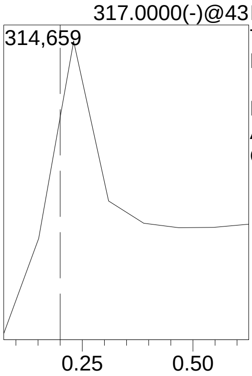

ID#:43 m/z: 317.0000
Type:Target
Name:Myricetin

Ret.Time:0.000
Area:0
Conc.:N.D.(Peak)

285.0000(-)@44

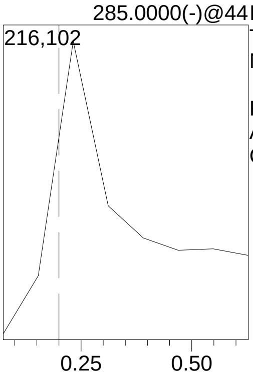

ID#:44 m/z: 285.0000
Type:Target
Name:Kaempeferol

Ret.Time:0.000
Area:0
Conc.:N.D.(Peak)

609.0000(-)@45

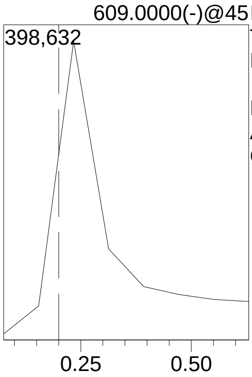

ID#:45 m/z: 609.0000
Type:Target
Name:rutin

Ret.Time:0.000
Area:0
Conc.:N.D.(Peak)

579.0000(-)@46

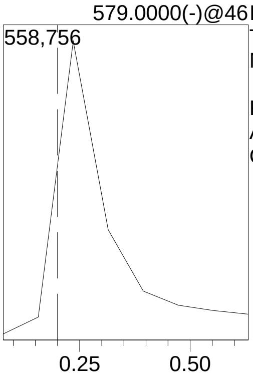

ID#:46 m/z: 579.0000
Type:Target
Name:narigin

Ret.Time:0.000
Area:0
Conc.:N.D.(Peak)
